# *VdGAL4* Modulates Microsclerotium Formation, Conidial Morphology, and Germination To Promote Virulence in Verticillium dahliae

**DOI:** 10.1128/spectrum.03515-22

**Published:** 2022-12-07

**Authors:** Yu Wen, Jinglong Zhou, Hongjie Feng, Wanqing Sun, Yalin Zhang, Lihong Zhao, Yong Cheng, Zili Feng, Heqin Zhu, Feng Wei

**Affiliations:** a Zhengzhou Research Base, State Key Laboratory of Cotton Biology, Zhengzhou University, Zhengzhou, China; b State Key Laboratory of Cotton Biology, Institute of Cotton Research of Chinese Academy of Agricultural Sciences, Anyang, China; Beijing Forestry University

**Keywords:** *Verticillium dahliae*, glycoside hydrolases, conidia, microsclerotia, pathogenicity, glycosidase hydrolases

## Abstract

Verticillium dahliae Kleb is a typical soilborne pathogen that can cause vascular wilt disease on more than 400 plants. Functional analysis of genes related to the growth and virulence is crucial to revealing the molecular mechanism of the pathogenicity of V. dahliae. Glycosidase hydrolases can hydrolyze the glycosidic bond, and some can cause host plant immune response to V. dahliae. Here, we reported a functional validation of VdGAL4 as an α-galactosidase that belongs to glycoside hydrolase family 27. VdGAL4 could cause plant cell death, and its signal peptide plays an important role in cellular immune response. VdGAL4-triggered cell death depends on BAK1 and SOBIR1 in Nicotiana benthamiana. In V. dahliae, the function of *VdGAL4* in mycelial growth, conidia, microsclerotium, and pathogenicity was studied by constructing *VdGAL4* deletion and complementation mutants. Results showed that the deletion of *VdGAL4* reduced the conidial yield and conidial germination rate of V. dahliae and changed the microscopic morphology of conidia; the mycelia were arranged more disorderly and were unable to produce microsclerotium. The *VdGAL4* deletion mutants exhibited reduced utilization of different carbon sources, such as raffinose and sucrose. The *VdGAL4* deletion mutants were also more sensitive to abiotic stress agents of SDS, sorbitol, low-temperature stress of 16°C, and high-temperature stress of 45°C. In addition, the *VdGAL4* deletion mutants lost the ability to penetrate cellophane and its mycelium were disorderly arranged. Remarkably, *VdGAL4* deletion mutants exhibited reduced pathogenicity of V. dahliae. These results showed that *VdGAL4* played a critical role in the pathogenicity of V. dahliae by regulating mycelial growth, conidial morphology, and the formation of microsclerotium.

**IMPORTANCE** This study showed that α-galactosidase *VdGAL4* of V. dahliae could activate plant immune response and plays an important role in conidial morphology and yield, formation of microsclerotia, and mycelial penetration. *VdGAL4* deletion mutants significantly reduced the pathogenicity of V. dahliae. These findings deepened the understanding of pathogenic virulence factors and how the mechanism of pathogenic fungi infected the host, which may help to seek new strategies for effective control of plant diseases caused by pathogenic fungi.

## INTRODUCTION

Verticillium dahliae Kleb is a soilborne plant-pathogenic fungus that can cause vascular wilt disease in more than 400 plants, including important economic crops and ornamental plants worldwide (1, 2). V. dahliae mainly exists in the forms of conidia, hyphae, and microsclerotia. Microsclerotia, the dormant survival structures of V. dahliae, can survive up to 14 years in the soil (3) and can resist extreme temperatures, desiccation, and other environmental stresses (4). The initial infection is usually caused by the germinated microsclerotia, which forms swelling hyphae and appressorium that directly penetrate the root and then colonize and grow in the xylem for a period of time, eventually leading to a series of typical symptoms of Verticillium wilt, such as wilting and yellowing of cotton leaves, browning of vascular bundle, and slow growth or even death of plants (5–7). Therefore, elucidating the growth and development and molecular mechanism of pathogenicity of V. dahliae is an important step to seeking new control strategies.

During the infestation of cotton by V. dahliae, a long-term battle pattern was formed between plants and pathogens. Plants employ defense systems to respond to pathogen attack. The first system is PAMP-triggered immunity (PTI), which is a basic defense that begins when plant cells recognize conserved pathogen-associated molecular patterns (PAMPs) through pattern recognition receptors (PRRs) (8). PRRs are membrane-localized leucine-rich proteins or kinases, such as brassinosteroid-insensitive 1-associated kinase (BAK1) and suppressor of BAK1-interacting receptor kinase 1 (SOBIR1) (9, 10). PTI mainly includes accumulation of reactive oxygen species and callose, electrolyte leakage, activation of mitogen-activated protein kinase (MAPK) pathway, and expression of plant defense genes (11). Pathogens’ cell wall-degrading enzymes act as virulence factors that induce plant immune responses, such as PAMPs (12, 13). However, the effector proteins secreted by pathogens can interfere with the recognition of PAMPs by host PRRs, thus blocking PTI immune response. The effector proteins are specifically recognized by the plant R protein and activate effector-triggered immunity (ETI). Fusarium oxysporum f. sp. *lycopersici* secreted the AvrII effector protein, which was a virulence factor recognized intracellularly by the tomato resistance protein, and induced host defense responses (12). Similarly, V. dahliae could use intracellular effector proteins to resist plant immune defense and successfully colonize the host plants (13).

In recent years, some of the critical genes in V. dahliae related to virulence have been characterized. V. dahliae *VdSsk1* was homologous to the Saccharomyces cerevisiae and was a response regulator of the two-component system; deletion of *VdSsk1* severely reduced the fungal virulence on tobacco seedlings, and *VdSsk1* was required for full virulence (14). A ubiquitin ligase (E3) enzyme, *VdBre1*, regulated mycelial growth, conidia production, lipid metabolism, and secondary metabolism of V. dahliae, and it played a key role in the process of cotton infection (15). *VdPR1* and *VdPR3* were involved in regulating the growth development and pathogenicity of V. dahliae (16, 17). Some genes not only regulate the vegetative growth of V. dahliae but also play important roles under a variety of abiotic stress conditions. The deletion mutant of RNA binding protein (RBP) *VdNop12* was more sensitive to low-temperature stress at 15°C, and *VdNop12* was essential for hyphal morphology, cold adaptation, and pathogenicity of V. dahliae (18). Deletion of the thiamine biosynthesis-related gene *VdTHI20* led to phenotypic defects and virulence damage in V. dahliae and increased tolerance to UV damage (19).

Microsclerotia and melanin are essential in the Verticillium wilt disease cycle. In recent years, several important genes related to the formation and development of microsclerotia and melanin have been identified. V. dahliae mitogen-activated protein, VMK1, played an important role in the development of microsclerotia, cell wall integrity, and Ca^2+^-signaling transduction (20). The fungal-specific transcription factor-encoding gene *Vdpf* played an important role in the formation of melanized microsclerotium and conidial production and pathogenicity, and the deletion mutants did not form microsclerotium and melanin, but they could make the mycelium transparent and inflate its end (21). V. dahliae *Vayg1*, a homolog of Exophiala dermatitidis (*Wayg1*) and Aspergillus fumigatus (*Aayg1*), was involved in production of microsclerotia and melanin biosynthesis and may catalyze two different precursors, one related to melanin synthesis (such as *YWA1* and *At4HN*), and the other was a key factor in the network pathway of microsclerotium formation (4).

Recent studies showed that a glycoside hydrolase family 12 (GH12) protein, XEG1, of Phytophthora sojae was involved in cellulose degradation and also acted as a PAMP to trigger cell death in several plant species, which, in turn, can be suppressed by effectors with an Arg-x-Leu-Arg motif (RXLR effectors) (22, 23). FoEG1, a secreted GH12 protein, contributed to the virulence of F. oxysporum according to its enzyme activity and acted as a PAMP to induce plant defense response (24). V. dahliae GH12 proteins EG1 and EG3 acted as PAMPs to trigger cell death and manipulated host immune responses (13). GH11 protein Vd424Y was a vital effector protein targeting the host nucleus, and it could regulate and activate immunity in plants (25). Although glycoside hydrolases had been studied in the immune effect of V. dahliae, there were few studies on GH27 proteins as virulence factors or effector proteins of V. dahliae (26).

Recently, VdGAL4 (VDAG_04757) in V. dahliae was identified as a pathogenicity-related protein by RNA-Seq analysis; its expression level in a high-virulent isolate was 22-fold higher than in a low-virulent isolate in quantitative reverse transcription PCR (RT-qPCR) analysis (S. Liu, R. Liu, Y. Wen, J. Zhou, H. Feng, Z. Feng, Y. Zhang, L. Zhao, H. Zhu, F. Wei, unpublished data). VdGAL4 was an alpha-galactosidase that belongs to GH27. VdGAL4 could cause plant cell death, which depended on the secretary function of signal peptide. VdGA4-triggered cell death depended on BAK1 and SOBIR1 in N. benthamiana. The deletion of *VdGAL4* led to changes in the morphology of conidia and mycelia of V. dahliae, inhibited the production of microsclerotia, and enhanced the resistance to abiotic stress. In addition, we obtained data that suggested that the *VdGAL4* deletion mutant also had reduced virulence of V. dahliae. Collectively, *VdGAL4* played a critical role in the fungal development and pathogenicity of V. dahliae as a virulence factor.

## RESULTS

### Identification of *VdGAL4* in V. dahliae.

An alpha-galactosidase gene (*VDAG_04757*) was identified in V. dahliae strain VdLs.17 (https://www.ncbi.nlm.nih.gov/genome/832), and it had a conserved glycosyl hydrolase family 27 (GH27) domain and was named *VdGAL4*. The full-length cDNA of *VdGAL4* was cloned from the strong pathogenic defoliating strain Vd080, which contained 1,215 bp and encoded a protein of 404 amino acids.

**FIG 1:**
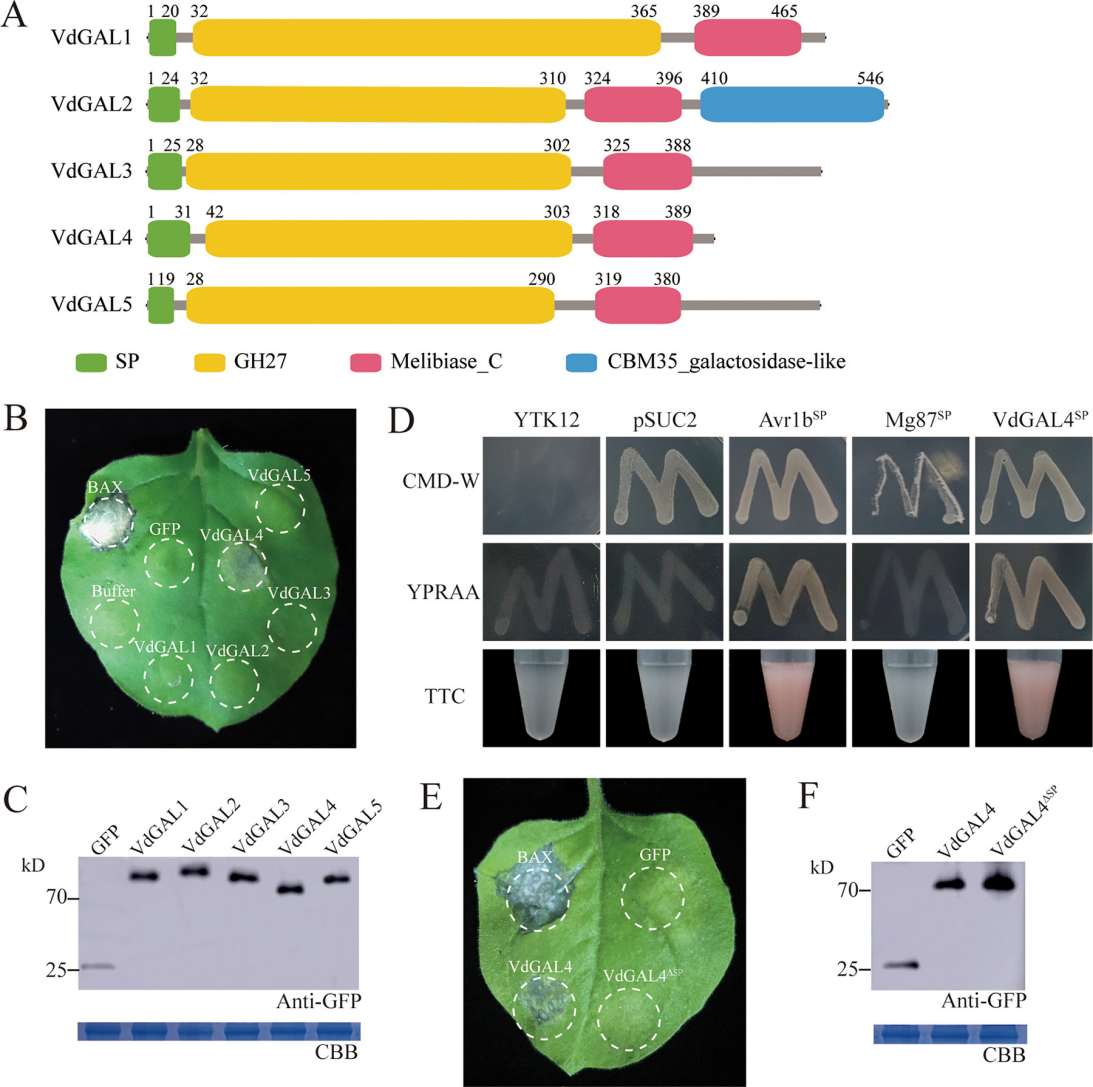
VdGAL4 can induce cell death, and the signal peptide (SP) is required for VdGAL4-induced cell death. (A) Schematics of the five GH27 proteins in V. dahliae and their domain structures. SP, signal peptide; Melibiase_C, Alpha galactosidase C-terminal beta-sandwich domain. (B) The cell death-inducing ability of GH27 proteins of V. dahliae and VdGAL4 induced cell death in Nicotiana benthamiana. The leaves of 4-week-old N. benthamiana were infiltrated with the indicated proteins. BAX and GFP were used as positive and negative controls, and the buffer was blank control (infected fluid). The photographs were taken 7 days postagroinfiltration (dpa). (C) Western blot analysis of transient expression proteins of GFP and VdGAL1-5. Proteins were stained with Coomassie brilliant blue (CBB) to determine equal loading. (D) Yeast signal trap system to verify the SP functions of VdGAL4. The known functional SPs of Avr1b and Mg87 were used as positive and negative controls, respectively. (E) The removed SP of VdGAL4 (VdGAL4^ΔSP^) could not cause cell death in N. benthamiana. (F) Western blot analysis of transient expression proteins of GFP, VdGAL4, and VdGAL4^ΔSP^.

From the bioinformatics analysis, five proteins (VdGAL1 to VdGAL5) with GH27 domain-containing proteins were identified in V. dahliae. Through prediction, it was found that each of these VdGAL proteins contains an N-terminal signal peptide, a GH27 domain, and a typical α-galactosidase C-terminal beta-sandwich domain (Melibiase_C), but only VdGAL4 had a transmembrane domain (Fig. 1A; see Fig. S1 in the supplemental material).

### VdGAL4 induced cell death in Nicotiana benthamiana depending on the secreted function of SP.

Agrobacterium tumefaciens-mediated transformation was used to transiently express each of the five proteins of GH27 in V. dahliae on N. benthamiana leaves. The sequences carrying *VdGAL1*, *VdGAL2*, *VdGAL3*, *VdGAL4*, and *VdGAL5* were cloned individually into the PVX vector pGR107. Interestingly, transient expression of these five genes showed that only VdGAL4 triggered typical cell death in N. benthamiana leaves 7 days after infiltration (Fig. 1B). Western blot analysis of the total protein extract from the agroinfiltrated area of the N. benthamiana leaves confirmed effective protein production by VdGAL1 to VdGAL5 (Fig. 1C). The results indicated that VdGAL4 can trigger cell death in N. benthamiana and may be involved in immunity manipulation in host-V. dahliae interactions.

To confirm the secreted characteristics of VdGAL4, signal peptide-mediated protein secretion was detected using the yeast signal trap system. SignalP5.0 prediction showed that the signal peptide (SP) of VdGAL4 was composed of 31 amino acids at the N terminus. The SPs of VdGAL4 were fused to the vector pSUC2 and generated pSUC2-VdGAL4^SP^. The recombined plasmid was transformed into the yeast YTK12. YTK12::pSUC2-VdGAL4^SP^ grew well in the YPRAA medium (2% raffinose) with raffinose as the sole carbon source, and the insoluble 2,3,5-triphenyltetrazolium chloride (TTC) could be reduced to insoluble red 1,3,5-triphenylformazan (TPF) (Fig. 1D). These results suggested that the SP of VdGAL4 had secretary characteristics and may be secreted into extracellular space under the guidance of its SP.

In addition, VdGAL4 may play a role in the pathogenesis of V. dahliae as a virulence factor. We found that VdGAL4^ΔSP^ could not induce cell death in N. benthamiana, which indicated that SP was required for VdGAL4 to induce cell death and VdGAL4 must target the extracellular space to induce cell death (Fig. 1E). Western blot analysis of the total protein extract from the agroinfiltrated area of the N. benthamiana leaves confirmed effective protein production by VdGAL4^ΔSP^ (Fig. 1F).

To further detect the subcellular location of *VdGAL4* in host cells, the *VdGAL4* sequences were fused with red fluorescent protein (RFP) (PBI121-VdGAL4-RFP) and transiently expressed in N. benthamiana leaves. The result showed that VdGAL4 was located on the cell membrane and nucleus of N. benthamiana (Fig. S2).

### NbBAK1 and NbSOBIR1 were required for VdGAL4-induced cell death in N. benthamiana.

**FIG 2:**
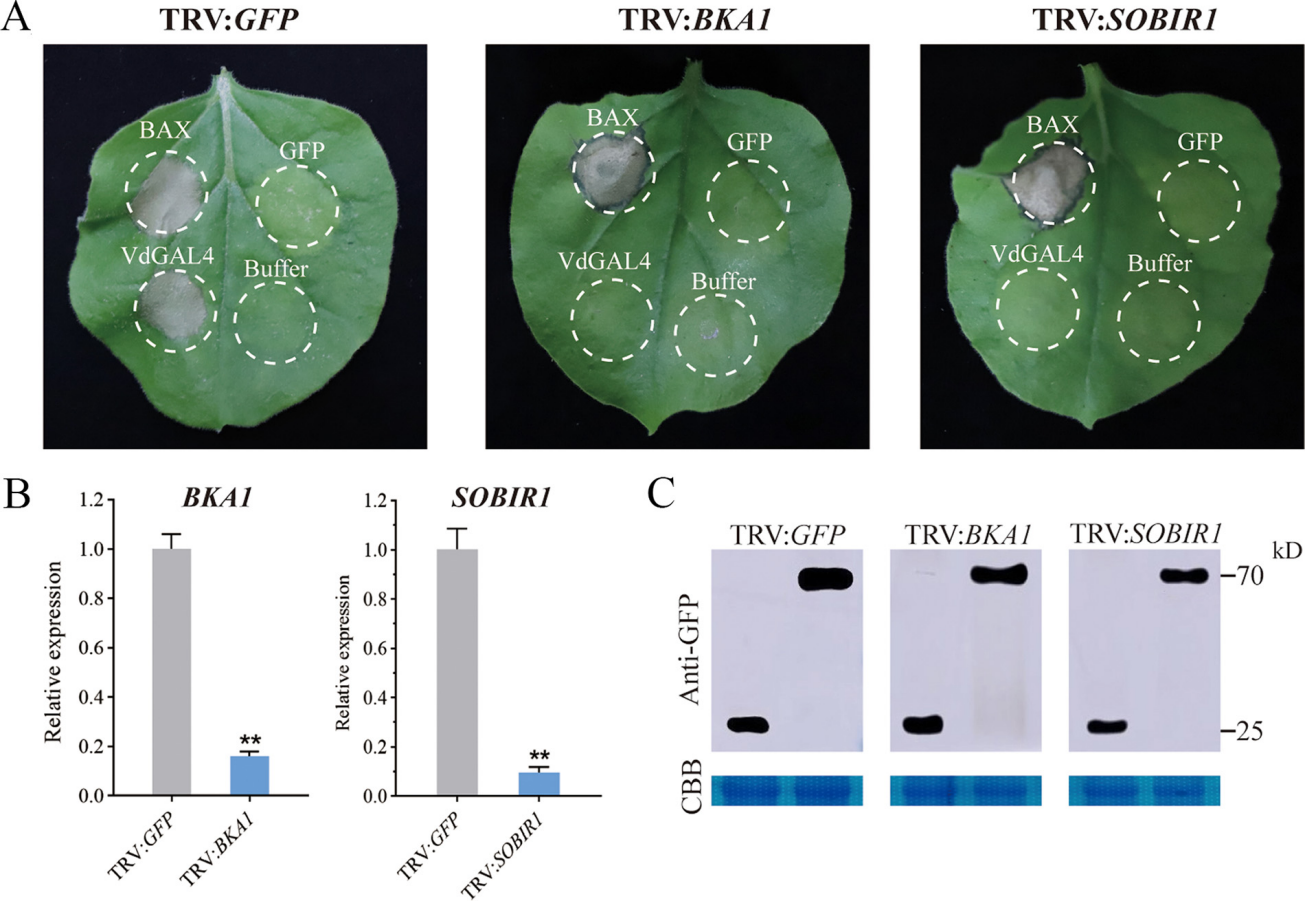
NbBAK1 and NbSOBIR1 were required for VdGAL4-induced cell death. (A) Virus-induced gene silencing (VIGS) assay was used to silence NbBAK1 and NbSOBIR1 by inoculation with TRV vectors (pTRV2-GFP, pTRV2-NbBAK1, pTRV2-NbSOBIR1) in N. benthamiana. Three weeks after inoculation, pGR107-VdGAL4, pGR107-BAX, and pGR107-GFP were transiently expressed in NbBAK1- and NbSOBIR1-silenced N. benthamiana leaves. The photographs were taken 7 dpa. (B) Expression levels of NbBAK1 and NbSOBIR1 after VIGS treatment by RT-qPCR. *NbActin* was used as the internal reference gene. (C) Western blot analysis of transient expression proteins of VdGAL4. Proteins were stained with Coomassie brilliant blue (CBB) to determine equal loading. **, *P* < 0.01.

VdGAL4-induced cell death was SP dependent, so we hypothesized that this induction might be BAK1/SOBIR1 dependent. We performed virus-induced gene silencing (VIGS) to silence NbBAK1 and NbSOBIR1 in N. benthamiana leaves and confirmed the silencing of NbBAK1 and NbSOBIR1 3 weeks after virus inoculation, and the expression of two genes was significantly decreased (Fig. 2B). In addition, the plants were agroinfiltrated with pGR107-VdGAL4, pGR107-Bcl-2-associated X protein (BAX), and pGR107-green fluorescent protein (GFP) vectors for transient expression. These results showed that the BAX induced cell death in *BAK1*, *SOBIR1*, and GFP-silenced N. benthamiana leaves at 7 dpi, while VdGAL4 did not trigger cell death on the same leaves of *BAK1*- and *SOBIR1*-silenced N. benthamiana (Fig. 2A). Immunoblotting confirmed that VdGAL4 was successfully expressed in N. benthamiana silenced with TRV2-NbBAK1, TRV2-NbSOBIR1, and TRV2-GFP (Fig. 2C). Thus, these results indicated that the plant cell death induced by VdGAL4 from V. dahliae was dependent on the receptor-associated kinases BAK1 and SOBIR1.

### Generation of *VdGAL4* mutants.

In order to study the function of *VdGAL4*, deletion mutants were constructed by replacing the coding sequence of *VdGAL4* in the wild-type (WT) strain Vd080 with a hygromycin resistance cassette via homologous recombination. After PCR verification, two independent deletion transformants were obtained, and Southern hybridization was used to verify that *Hyg* was a single copy in the two deletion mutants (Fig. S3). Then, the genome sequence and promoter region of *VdGAL4* were reintroduced into the deletion mutants, and the corresponding complementary mutant strains were obtained by PCR verification (Fig. S3).

### Enzyme activity and hydrolytic activity of VdGAL4.

**FIG 3:**
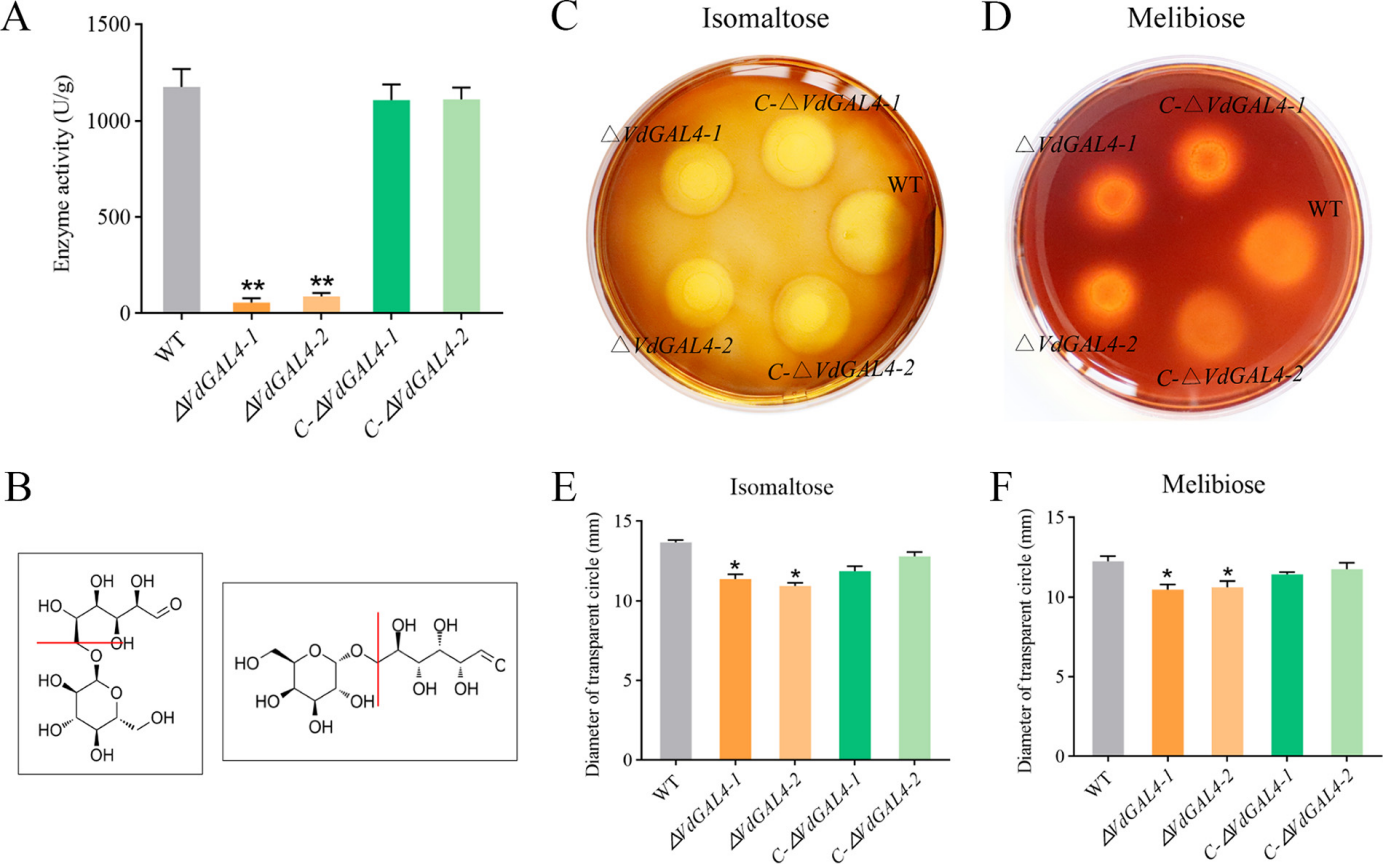
Enzyme activity and hydrolytic activity of VdGAL4. (A) α-Galactosidase activity of wild-type (WT) Verticillium dahliae strain Vd080, the two *VdGAL4* deletion mutant strains (*ΔVdGAL4-1* and *ΔVdGAL4-2* strains), and the two *VdGAL4* complement mutant strains (*C-ΔVdGAL4-1* and *C-ΔVdGAL4-2* deletion mutant strains). (B) Structural formulas of isomaltose and melibiose and the cleavage sites of α-1,6 glycosidic bonds of VdGAL4. (C, D) VdGAL4 hydrolyses isomaltose (C) and melibiose (D); the transparent circles of all tested strains were observed by iodine-KI staining solution. (E, F) Diameter of transparent circles on water agar medium amended with isomaltose (E) and melibiose (F). The error bar represents standard error of the mean. *, *P* < 0.05; **, *P* < 0.01.

To analyze α-galactosidase activity of VdGAL4, the α-galactosidase activity of the WT strain reached 1,175.48 ± 93.56 U/g. However, the α-galactosidase activity levels of *ΔVdGAL4* strains (*ΔVdGAL4-1* and *ΔVdGAL4-2* deletion mutant strains) were only 57.04 ± 21.46 U/g and 87.60 ± 18.67 U/g, respectively, and were only 4.85% to 7.40% of that of the WT strain (Fig. 3A).

Since isomaltose and melibiose contain α-1,6 glycosidic bonds (Fig. 3B), we determined the ability of VdGAL4 to hydrolyze α-1,6 glycosidic bonds by measuring the hydrolysis ability of different mutants to these two disaccharides. The transparent circle produced by *ΔVdGAL4* strains was significantly smaller than the WT and *C-ΔVdGAL4* strains in the water agar medium containing isomaltose and melibiose, respectively (Fig. 3C and D). The diameters of Δ*VdGAL4* strains in the water agar medium amended with isomaltose were 11.38 ± 0.29 mm (Δ*VdGAL4-1*) and 10.94 ± 0.20 mm (Δ*VdGAL4-1*), which were significantly lower than WT and C-Δ*VdGAL4* strains; the same was true in the water agar medium amended with melibiose with the corresponding values of 10.47 ± 0.34 mm and 10.62 ± 0.40 mm (Fig. 3E and F). These results showed that deletion mutants reduced the hydrolysis activity of isomaltose and melibiose, further indicating that VdGAL4 could efficiently hydrolyze α-1,6 glycosidic bonds.

### *VdGAL4* was indispensable for normal vegetative growth of mycelium and conidia.

**FIG 4:**
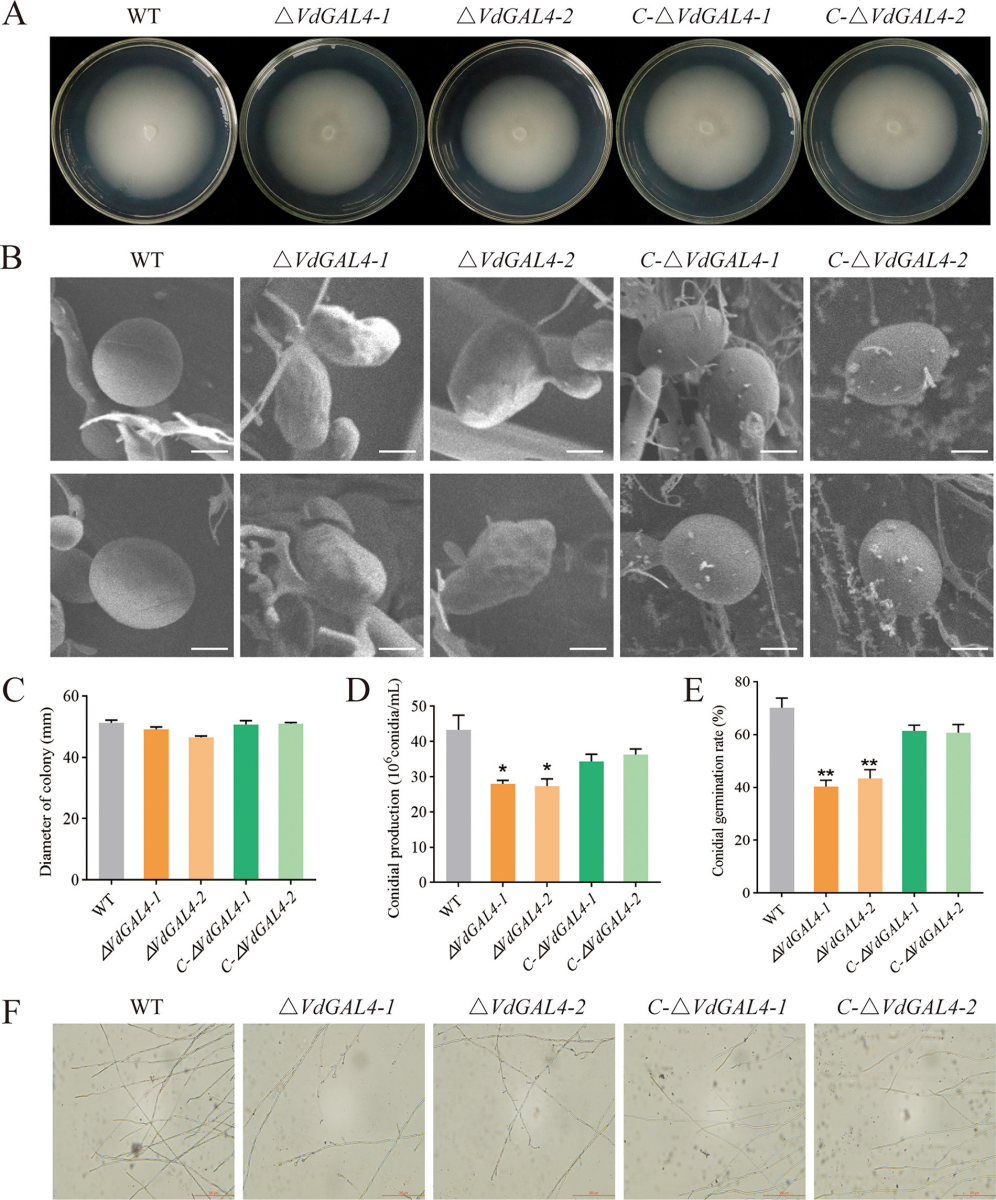
Assays of the role of *VdGAL4* in the normal vegetative growth and conidial production of V. dahliae. (A) Colony morphology of WT, *ΔVdGAL4*, and *C-ΔVdGAL4* strains in PDA at 25°C for 14 days. (B) Morphology of the conidia of all tested strains under scanning electron microscope. Bar, 5 μm. (C) Growth diameter of all tested strains shown in panel A. (D) The conidial production of all tested strains after 5 days on Czapek-Dox medium. (E) Conidial germination rate of all tested strains. (F) Mycelium morphology of all tested strains under light microscope. Bar, 200 μm. The error bar represents standard error of the mean. *, *P* < 0.05; **, *P* < 0.01.

After being cultured on potato-dextrose agar (PDA) medium at 25°C for 14 days, the phenotypes of the *ΔVdGAL4* strains (*ΔVdGAL4-1* and *ΔVdGAL4-2*) were similar to that of the WT and *C-ΔVdGAL4* strains (*C-ΔVdGAL4-1* and *C-ΔVdGAL4-2*), and there was no significant difference in radial growth diameter (Fig. 4A and C).

The conidia of the WT and *C-ΔVdGAL4* strains were plump and smooth, while the conidia of the *ΔVdGAL4* strain showed shrinkage with dents and irregular shapes on the surface (Fig. 4B). By comparing conidial production, the *ΔVdGAL4* strain was significantly lower than WT and *C-ΔVdGAL4* strains (Fig. 4D). Under normal culture conditions, the germination rate of WT conidia reached 70.21%, which was similar to that of the *C-ΔVdGAL4* strain, while the germination rate of the *ΔVdGAL4* strain was only 40.35% to 43.45%, showing significant differences (Fig. 4E; Fig. S4A). In addition, the relative expression levels of some genes related to the conidial production, including *VdNLP1*, *VdPLP*, and *Vdpf*, were markedly downregulated in *ΔVdGAL4* strains compared with the WT and *C-ΔVdGAL4* strains (Fig. S4B). It is further explained that there is an important role of *VdGAL4* in the conidial production of V. dahliae.

The microscopic morphology of mycelium was observed by microscope, and the hyphal growth of WT and *C-ΔVdGAL4* strains was relatively regular, while the hyphal growth of *ΔVdGAL4* strains had more branches (Fig. 4F). These results showed that *VdGAL4* was necessary for normal vegetative growth of mycelium.

### *VdGAL4* affected the production of microsclerotium and melanin.

**FIG 5:**
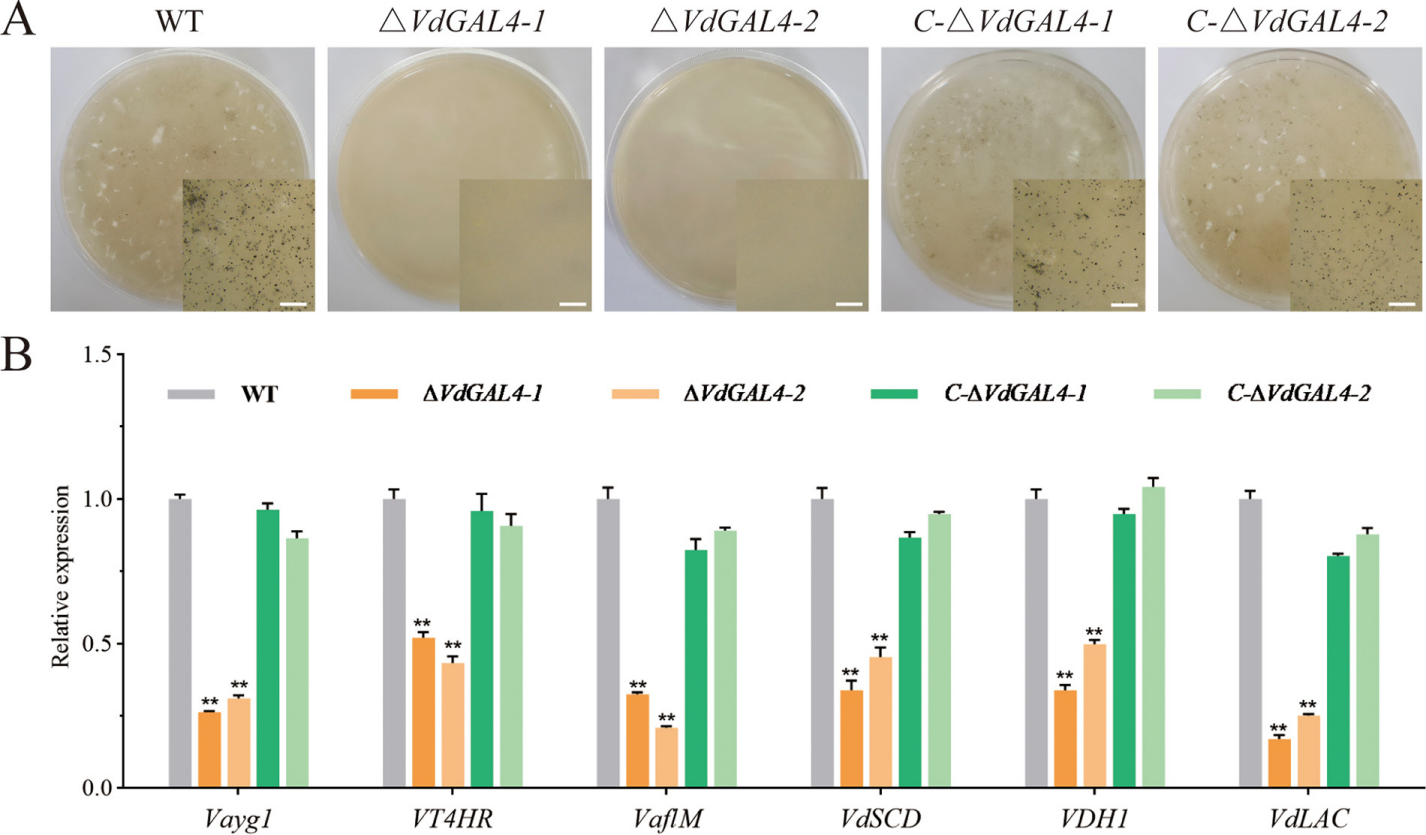
Assays of the role of *VdGAL4* in the formation of microsclerotium and melanin. (A) Microsclerotium formation of WT, *ΔVdGAL4*, and *C-ΔVdGAL4* strains cultured in BMM medium for 40 days. The microsclerotium in the right bottom corner is shown by microscopy. Bar, 1 mm. (B) Assays of the relative expression of melanin-related genes by RT-qPCR. The error bar represents standard error of the mean. **, *P* < 0.01.

After incubation on basal modified medium (BMM) medium for 40 days, cultures of WT and *VdGAL4* deletion and complementary strains were assessed under a microscope to monitor microsclerotium development. No microsclerotia were observed for the *ΔVdGAL4* strains at 40 days. However, there were abundant, darkly pigmented microsclerotia in the WT and *C-ΔVdGAL4* strains (Fig. 5A), and the numbers of microsclerotium reached 314,270 and 286 per cm^2^, respectively.

The relative expressions of six genes related to the melanin, including *Vayg1*, *VT4HR*, *VaflM*, *VdSCD*, *VDH1*, and *VdLAC*, were significantly lower than the WT and *C-ΔVdGAL4* strains (Fig. 5B). Thus, *VdGAL4* was apparently involved in the production of melanin and microsclerotium in V. dahliae.

### The utilization efficiency of *VdGAL4* for different carbon sources was different.

**FIG 6:**
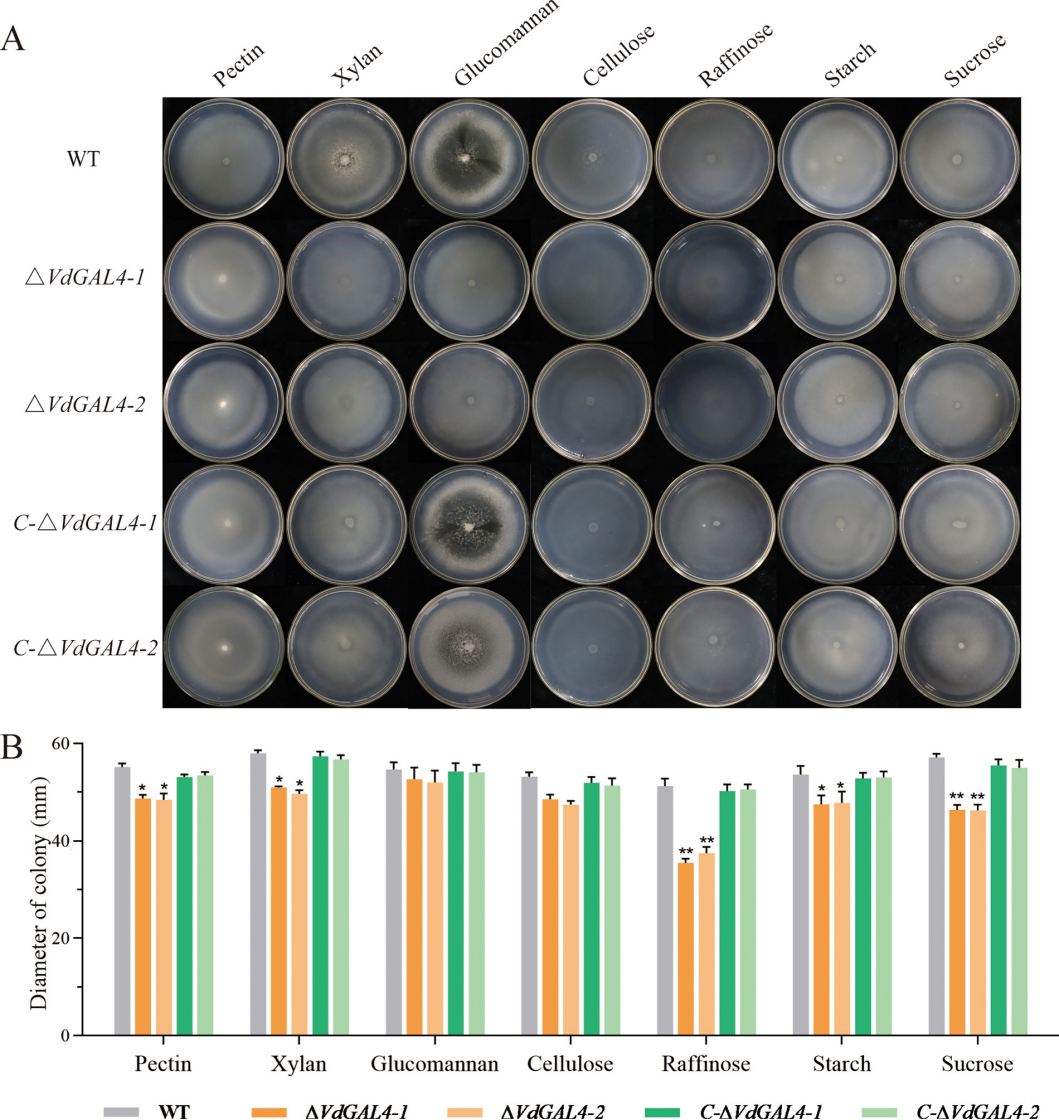
Assays of the role of *VdGAL4* in the utilization of different carbon sources. (A) WT, *ΔVdGAL4*, and *C-ΔVdGAL4* strains were cultured in Czapek-Dox medium amended with different carbon sources, pectin, xylan, glucomannan, cellulose, raffinose, starch, and sucrose at 25°C for 14 days. (B) Growth diameter of all tested strains shown in panel A. Error bar represents standard error of the mean. *, *P* < 0.05; **, *P* < 0.01.

To analyze the function of *VdGAL4* on mycelial growth and carbon utilization, the mycelial growth of the WT and mutant strains was measured in Czapek-Dox medium amended with different carbon sources (pectin, xylan, glucomannan, cellulose, raffinose, starch, and sucrose) (Fig. 6A). After incubation in Czapek-Dox agar medium for 14 days, *ΔVdGAL4* strains displayed a significant inhibition of colony growth in medium amended with different carbon sources except for glucomannan and cellulose. Especially in the presence of raffinose and sucrose, the relative growth of *ΔVdGAL4* strains was reduced from 26.90% to 30.76% and 16.91% to 18.80% compared with WT strains (Fig. 6B). These results indicated that *VdGAL4* was important for vegetative growth of V. dahliae and carbon source utilization ability of raffinose and sucrose.

### Resistance of *ΔVdGAL4* strains to different stresses.

**FIG 7:**
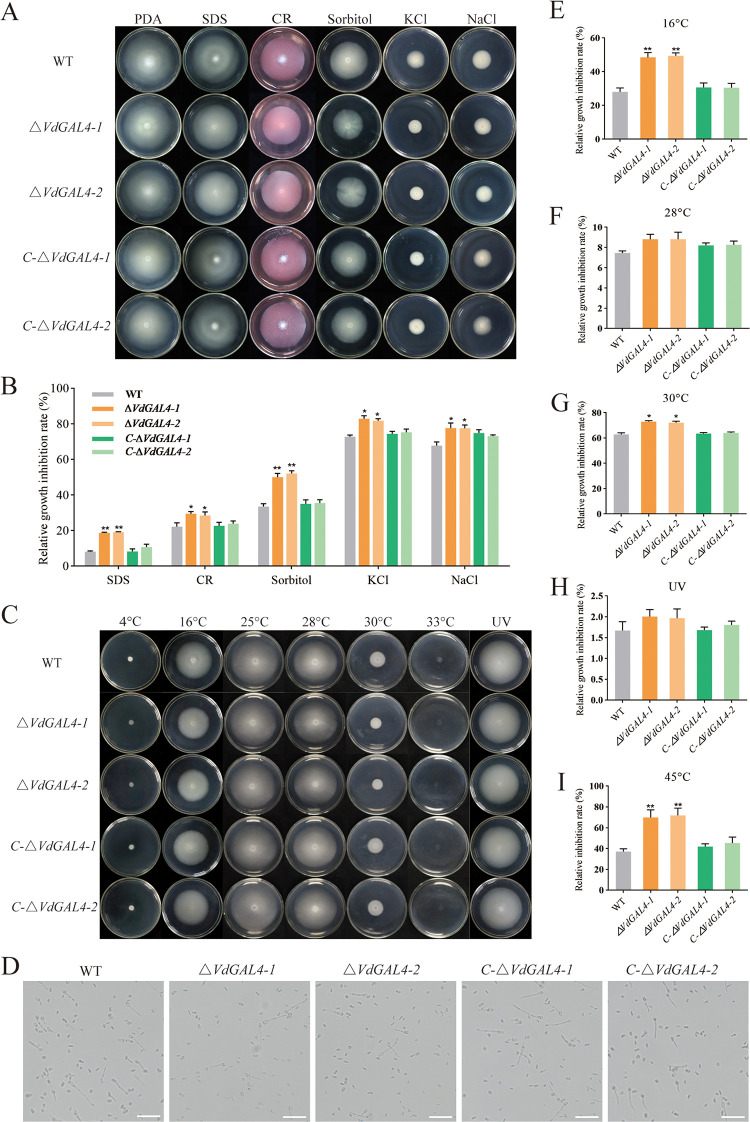
Assays of the role of *VdGAL4* in resistance to abiotic stresses. (A) WT, *ΔVdGAL4*, and *C-ΔVdGAL4* strains were cultured at 25°C for 14 days in PDA medium supplemented with 0.002% SDS, 0.02% Congo red, 1 M sorbitol, 1 M KCl, and 1 M NaCl. (B) Relative growth inhibition rate of colony growth. (C) The colony morphology of the tested strains was cultured in PDA medium treated at 4°C, 16°C, 25°C, 28°C, 30°C, 33°C, or with 10 s UV for 14 days. (D) Conidia of the tested strains germinated at 45°C for an hour. Bar, 200 μm. (E to G) Relative growth inhibition rate of colony growth at 16°C (E), 28°C (F), and 30°C (G). (H) Relative growth inhibition rate of colony growth under UV irradiation. (I) Relative inhibition rate of conidial germination rate of the tested strains at 45°C. The error bar represents standard error of the mean. *, *P* < 0.05; **, *P* < 0.01.

There was no significant difference among the diameters of WT, *ΔVdGAL4*, and *C-ΔVdGAL4* strains in PDA for 14 days (Fig. 7A), while the relative growth inhibition rate (RGI) of *ΔVdGAL4* strains was significantly higher than WT and *C-ΔVdGAL4* strains under all stress conditions (Fig. 7B). Especially under stress conditions of SDS and sorbitol, the RGIs of *ΔVdGAL4* strains were increased from 129.39% to 134.15% and 49.84% to 56.04% compared to WT strains. These results suggested that *VdGAL4* was more sensitive to abiotic stress agents of SDS and sorbitol, indicating that *VdGAL4* had a more obvious response to the cell membrane and cell wall.

In addition, we determined the sensitivity of *VdGAL4* to cold, heat, and UV stress. There was no significant change in the RGI of all strains at 25°C, 28°C, and UV (Fig. 7F and H). At 4°C and 33°C, almost all the tested strains could not grow. However, under low-temperature stress of 16°C, the RGIs of the *ΔVdGAL4-1* and *ΔVdGAL4-2* strains reached 48.54% and 49.42%, respectively, which were significantly higher than WT and *C-ΔVdGAL4* strains (Fig. 7E). Under heat temperature stress of 30°C, the RGIs of the *ΔVdGAL4-1* and *ΔVdGAL4-2* strains reached 72.94% and 72.10%, respectively, which were significantly different from WT and *C-ΔVdGAL4* strains (Fig. 7G). Also, we measured the thermal stability of *VdGAL4* at 45°C and found that the relative inhibition rate of the conidia germination rate of *ΔVdGAL4* strains was significantly lower than WT and *C-ΔVdGAL4* strains (Fig. 7D and I). These results suggested that *VdGAL4* was more sensitive to low-temperature stress of 16°C and high-temperature stress of 30°C, and the conidia germination of the *ΔVdGAL4* strain is more sensitive to high temperature of 45°C.

### *VdGAL4* was essential for mycelium penetration of V. dahliae.

**FIG 8:**
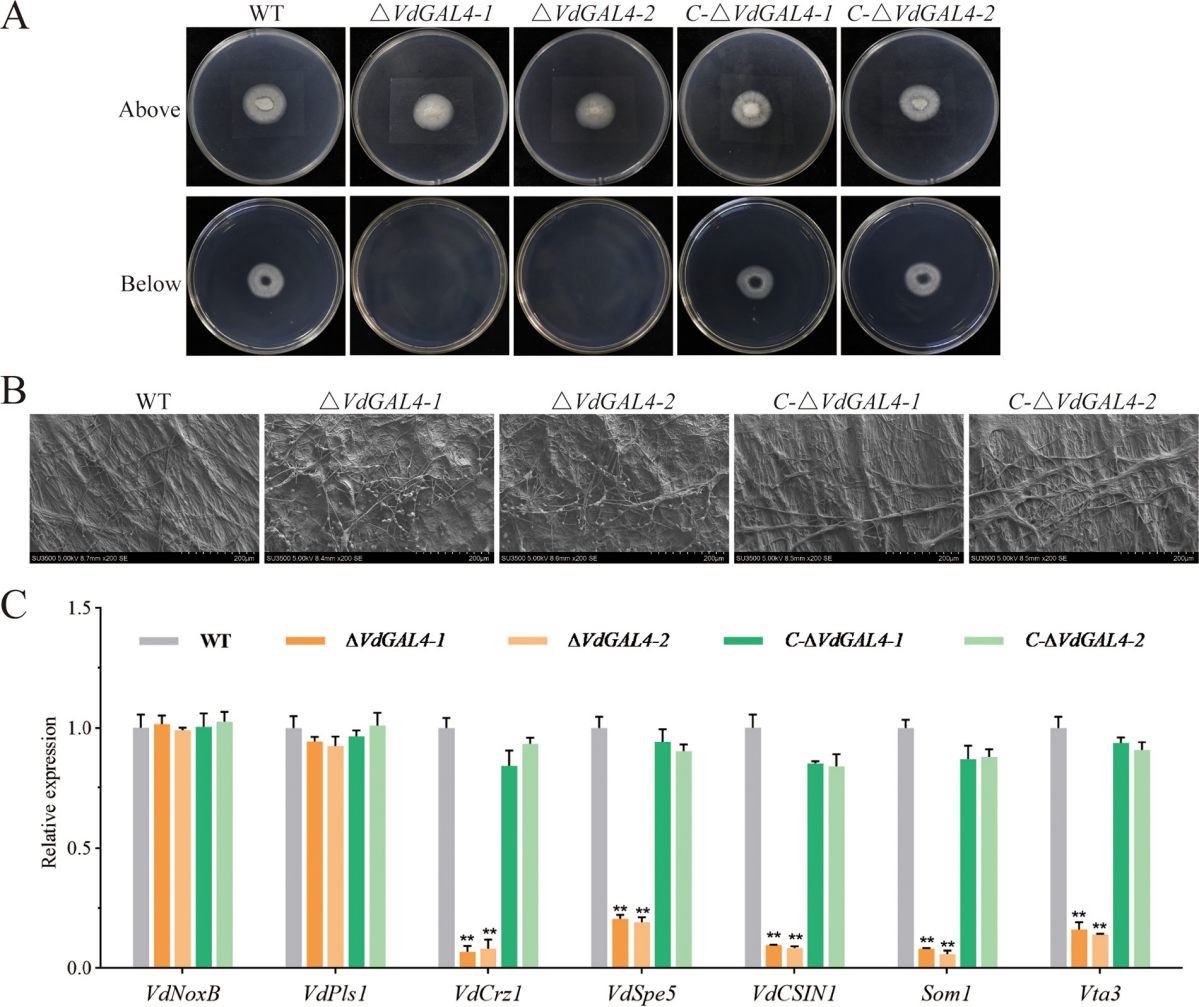
Assays of the role of *VdGAL4* in penetration of mycelium. (A) Cellophane membranes were plated onto PDA and inoculated with conidia suspension from WT, *ΔVdGAL4*, and *C-ΔVdGAL4* strains and grown on cellophane for 3 days. (A, Bottom) Indicated cellophane was removed for 3 days. (B) Micromorphology of mycelium of all tested strains observed on cellophane by scanning electron microscope. Bar, 200 μm. (C) Assays of the relative expression of conidia-related genes in all tested strains by RT-qPCR. The error bar represents standard error of the mean. **, *P* < 0.01.

The effect of *VdGAL4* on the penetration ability of mycelium was tested by cellophane penetration test. Three days after the cellophane was removed, the mycelia of WT and *C-ΔVdGAL4* strains could penetrate the cellophane and continue to grow in the PDA medium, while the *ΔVdGAL4* strains could not penetrate the cellophane (Fig. 8A).

The microscopic morphology of mycelium on cellophane was observed by scanning electron microscope; the hyphal growth of WT and *C-ΔVdGAL4* strains was relatively normal and regular, while the mycelium growth of *ΔVdGAL4* strains was disordered and branched (Fig. 8B). These results showed that *VdGAL4* was necessary to penetrate the host plant root.

In addition, there was no significant change in the expression of infection peg development related genes *VdNoxB* and *VdPls1* in *ΔVdGAL4*, WT, and *C-ΔVdGAL4* strains. However, the relative expression of some genes related to the infection peg development, appressorium formation, penetration, and colonization in plant roots, including *VdCrz1*, *VdSep5*, VdCSIN1, *Som1*, and *Vta3*, was significantly downregulated in *ΔVdGAL4* strains compared with the WT and *C-ΔVdGAL4* strains (Fig. 8C).

### *VdGAL4* was essential for full virulence in V. dahliae on cotton.

**FIG 9:**
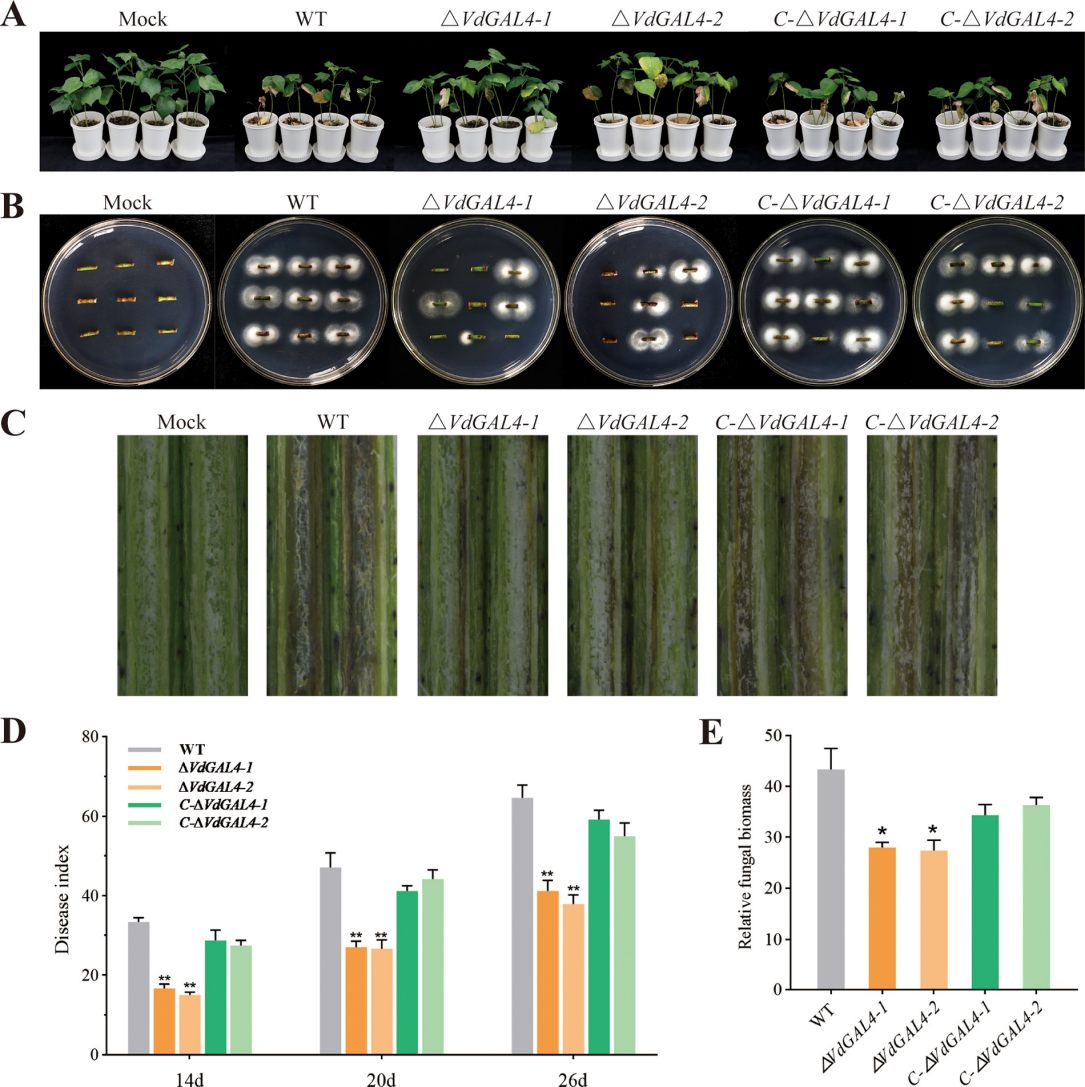
Verticillium dahliae *VdGAL4* was a virulence factor in cotton. (A) Cotton seedlings (Gossypium hirsutum cv. Jimian11) were mock inoculated (Mock) or inoculated with WT, *ΔVdGAL4*, and *C-ΔVdGAL4* strains and photographed at 26 days postinoculation. (B) V. dahliae from the cotton stem was reisolated on PDA medium at 25°C for 5 days. (C) Observation of browning of vascular bundle in the longitudinal section of stem of cotton seedlings. (D) Disease index of cotton seedlings inoculated with all tested strains at 14, 20, and 26 days postinoculation. (E) Colonization, by RT-qPCR, of V. dahliae in cotton stem after 26 days of infection. The error bar represents standard error of the mean. *, *P* < 0.05; **, *P* < 0.01.

At 26 days postinoculation (dpi), mock-inoculated cotton seedlings were growing well without any disease symptoms, while cotton seedlings inoculated with the WT and *C-ΔVdGAL4* strains showed typical Verticillium wilt symptoms, including wilting, necrosis, and vascular bundle browning (Fig. 9A to C). The disease index of plants inoculated with *ΔVdGAL4* strains was significantly less than plants inoculated with the WT and *C-ΔVdGAL4* strains (Fig. 9D). The disease index of cotton seedlings inoculated with the WT strain reached 64.58 ± 5.64, while *ΔVdGAL4-1* and *ΔVdGAL4-2* strains were 41.25 ± 4.51 and 37.92 ± 4.02, respectively, and the browning degree of vascular bundles was also significantly reduced (Fig. 9C and D). Furthermore, fewer fungal colonies grew from excised stem sections of seedlings inoculated with the *ΔVdGAL4* strains than with WT and *C-ΔVdGAL4* strains (Fig. 9B). Quantification of fungal biomass by qPCR confirmed that the fungal biomass of cotton stems inoculated with WT and *C-ΔVdGAL4* strains was about twice as much as that the *ΔVdGAL4* strains (Fig. 9E). Therefore, the *ΔVdGAL4* strains significantly weakened the infection of V. dahliae to cotton and indicated that *VdGAL4* was crucial to the virulence of V. dahliae.

## DISCUSSION

α-Galactosidase (α-Gal; EC 3.2.1.22) is widely present in plants, animals, and microorganisms, including F. oxysporum, Trichoderma reesei, Pichia pastoris, and Aspergillus niger (27). In this study, a glycoside hydrolase family 27 α-galactosidase was identified from V. dahliae. Glycoside hydrolases (GH, EC 3.2.1) are enzymes that hydrolyze glycosidic bonds and play an important role in the hydrolysis and synthesis of sugars and glycoconjugates in organisms. Previous studies have shown that the different GH family members (*FoEG1*, *PsXEG1*, *VdXyn4*, *Vd424Y*, *VdEG1*, and *VdEG3*) in different plant pathogens can cause plant cell necrosis through different ways, stimulate plant defense response, and have different effects on pathogenicity (13, 22, 24, 25, 28). In the present study, we found VdGAL4 had the ability to trigger cell death and plant immunity, and the protein played a critical role in fungal pathogenicity as a virulence factor during infection and colonization.

Fungal pathogens can regulate plant immune response by secreting effector proteins (27). There are many effector proteins in V. dahliae that have been identified, such as the hydrophobin VdHP1, that can cause plant cell death and act as an effector to induce plant immunity (29). The CFEM members VdSCP76 and VdSCP77 played a key role in immune regulation and had a broad-spectrum ability to inhibit cell death (30). Recently, the secretary proteins of GH12 family FoEG1 of F. oxysporum and PsXEG1 of Phytophthora sojae acted as a pathogen-related molecular pattern (PAMP) on the extracellular matrix of plants to induce cell death (23, 24, 31). Xyloglucan-specific endoglucanase (XEG1) could induce plant defense responses in a BAK1-dependent manner, and the ability of FoEG1 to induce cell death was mediated by BAK1 and SOBIR1; this ability was independent of its hydrolase activity (23). Similarly, GH12 proteins VdEG1 and VdEG3 in V. dahliae triggered immunity through two different mechanisms and cooperated with novel effectors to manipulate immunity during N. benthamiana infection; BAK1 is required for VdEG1- and VdEG3-triggered immunity, while SOBIR1 is specifically required for VdEG1-triggered immunity in N. benthamiana (13, 22). Vd424Y also can induce cell death in many plant species. The transient expression of GH11 family protein Vd424Y in N. benthamiana induced BAK1- and SOBIR1-dependent cell death (25). In this study, unlike the other four proteins in the GH27 family of V. dahliae, VdGAL4 can induce cell death and hypersensitive response (HR) in N. benthamiana, and VdGAL4 SP can secrete invertase in yeast and transfer VdGAL4 into the extracellular space (Fig. 1 and 2). Also, VdGA4-triggered cell death was dependent on BAK1 and SOBIR1 in N. benthamiana, which indicated that VdGAL4 can act as a secreted protein to activate the immune response of plants.

α-Galactosidase is an exoglycosidase that catalyzes the hydrolysis of α-galactosidase bonds and can specifically catalyze the hydrolysis of α-1,6 glycosidic bonds (32, 33). α-Galactosidase is mainly involved in the degradation of raffinose, stachyose, melibiose, and galactomannan (34, 35). In this study, the content of α-galactosidase in V. dahliae decreased significantly after deletion of *VdGAL4*. In the *VdGAL4* deletion mutants, the content of α-galactosidase was reduced, and the ability of *VdGAL4* to hydrolyze α-1,6 glycosidic bonds was also decreased significantly. Therefore, the degree of hydrolyzing isomaltose and melibiose by the *ΔVdGAL4* strain was also weakened significantly (Fig. 3). In addition, galactosidase can catalyze the production of d-galactose and rapidly convert and consume it through the glycolysis pathway (36), providing the initial energy source for plant seed germination and participating in the hydrolysis of polysaccharides stored in the cell wall (37).

Conidia and mycelium played an important role in the infection process and pathogenic cycle of V. dahliae (38, 39). In recent years, some genes involved in vegetative growth and/or pathogenicity of V. dahliae have been identified, as well as some resistance genes, including induction of defense signaling pathways and resistance to V. dahliae infection (39). *VDH1* encoded a hydrophobic protein, which regulates the development of conidia and hyphae, enhances the dehydration tolerance of conidia, and plays an important role in the formation of microsclerotium (40, 41). The corresponding knockout strains of *VdNLP1* and *VdNLP2* produced fewer conidia but more vigorous aerial mycelium (42). However, *VdGRP1* inhibited mycelial growth and promoted microsclerotium formation under normal conditions (43). In the present study, the deletion of *VdGAL4* reduced the conidia yield and conidia germination of V. dahliae and affected the microscopic morphology of conidia. In PDA medium, there were no significant changes in the aerial mycelium and radial growth diameter between the *VdGAL4* deletion mutants and WT strain; however, under the microscope, it was found that the mycelia of *VdGAL4* deletion mutants were disordered and branched, and their growth was inhibited (Fig. 4).

Some fungi, including V. dahliae, can produce 1,8-dihydroxynaphthalene (DHN)-melanin during microsclerotium formation and maturation, which helps them resist various environmental stresses and is important for the long-term survival of the pathogen (26, 44, 45). *Vayg1* was required for microsclerotium formation and melanin production in V. dahliae (4). *VdPKS9* and *VdMRTF1* were negative transcription regulators for melanin biosynthesis, microsclerotium formation, and virulence in V. dahliae (46, 47). In contrast, we found that the *VdGAL4* deletion mutants could not produce microsclerotium in BMM medium, and the expression of genes related to melanin production was significantly downregulated (Fig. 5). Thus, we may conclude that *VdGAL4* is required for microsclerotium and melanin formation. However, further investigation is needed to elucidate the exact roles *VdGAL4* played in microsclerotium and melanin formation.

Many studies have shown that some genes in V. dahliae played a role in stress resistance and tolerance and participated in regulating the growth and development of V. dahliae, thus affecting the infection and pathogenicity of V. dahliae in plants (14, 29, 48, 49). Plant cell walls have the function of resisting the infection and stress of pathogens. The infection and colonization of pathogenic fungi are usually related to the degradation of host cell walls (50). The initial stage of decomposition is usually dominated by the colonization of pathogen-expressing enzymes that can attack the easily accessible cell wall components, such as hemicelluloses and pectins (51). The inner surface of the cell wall deposits into the secondary cell wall, including cellulose, lignin, and xylan (52). The study on the effect and utilization of cell wall components is helpful to understand the mechanism of plant cell wall damage caused by pathogens (53, 54). Pyricularia oryzae glycerol-3-phosphate dehydrogenase *PoGpd*, a cellular oxidoreductase, played an important role in carbon source utilization, mycelial growth, and virulence (55). The α-oxoglutarate dehydrogenase *VdOGDH* deletion mutants grew slowly in the medium with sucrose, pectin, xylan, starch, and galactose as the sole carbon source, indicating that *VdOGDH* was important for vegetative growth and carbon utilization of V. dahliae (56). Knockout of Cu-Zn superoxide dismutase *VdSOD1* did not alter vegetative growth and conidia production under normal growth conditions, but it weakened cellulose utilization (57). In this study, α-galactosidase had hydrolytic activity to hydrolyze raffinose; *VdGAL4* deletion affected the utilization of different carbon sources, such as raffinose and sucrose (Fig. 6). The V. dahliae *VdSsk1* and *VdSsk2* deletion mutant showed more sensitivity to NaCl and sorbitol, while resistance to CR was significantly enhanced. *VdSsk1* and *VdSsk2* were involved in the response to various stresses, including high osmotic stress (14, 49). Cold is an adverse temperature condition that fungi often encounter in nature (58). *VdNop12* affected the sensitivity of V. dahliae to low-temperature stress, the conidia of Beauveria bassiana *hyd1* deletion mutant were more heat resistant, and the *VdThit* deletion mutant was more sensitive to UV stress (18, 59, 60). In this study, *VdGAL4* deletion mutants were more sensitive to abiotic stress agents of SDS, sorbitol, low-temperature stress of 16°C, and high-temperature stress of 33°C and 45°C (Fig. 7). These results indicated that V. dahliae *VdGAL4* may be involved in the process of plant infection by damaging the cell membrane and cell wall of hosts, and its resistance to stress is also indirect evidence of its virulence.

*VdHP1* did not affect mycelium penetration but also contributed to mycelium growth in the process of initial colonization of V. dahliae (29). However, the deletion of *VdGAL4* in V. dahliae led to loss of the ability to penetrate cellophane, and its mycelium arrangement was more disordered (Fig. 8). *VdNoxB*/*VdPls1* mediated ROS production and regulated the formation of penetration peg in the initial colonization of cotton roots (61). However, in this study, the deletion of *VdGAL4* did not affect the expression of the *VdNoxB* and *VdPls1*, indicating that *VdGAL4* may not affect the penetration peg structure of V. dahliae. Both nuclear transcription factors *Som1* and *Vta3* regulated developmental genetic networks required for formation of conidia and microsclerotium of V. dahliae (11). *VdCrz1* plays important roles in Ca^2+^ signaling, cell wall integrity, regulating infection peg development, and microsclerotia development and is also important for full virulence of V. dahliae (20). The expression of *VdCrz1*, *VdSep5*, *VdCSIN1*, *Som1*, and *Vta3* was significantly downregulated in *ΔVdGAL4* strains (Fig. 8). We speculated that although *VdGAL4* did not directly affect the formation of penetration pegs, it may indirectly affect the infection of V. dahliae to cotton by affecting the mycelium neck ring structure, penetration infection structure, and appressorium formation.

Results in this study also showed *VdGAL4* deficiency could significantly reduce the pathogenicity of V. dahliae to cotton, and the fungal colonization in the host is significantly reduced (Fig. 9). Previous research found the link between melanin, microsclerotia, and fungal virulence. *DHN*-melanin is essential for the penetration of Magnaporthe grisea to host plants and affects the ability of appressorium to penetrate plant leaves (62). *VdOCH1* encoded α-1,6-mannosyltransferase, manipulating the colony morphology and conidia, microsclerotium formation, and pathogenic ability of V. dahliae in sunflower (63). The mycelial growth and conidial formation of the *Vayg1* deletion mutant were reduced, while the resistance to oxidative stress was enhanced, and *Vayg1* was necessary for melanin and micronucleus formation, which may be the reason for its reduced virulence (4). Similarly, *VdGAL4* affects the pathogenicity of V. dahliae by regulating mycelial growth, conidial morphology, and the formation of microsclerotium. In addition, α-galactosidase can hydrolyze the glycosidic bond and then destroy the cell wall structure in the process of V. dahliae infecting plants, thus accelerating the infection and making the plants more seriously infected by pathogens.

In summary, the transient expression of VdGAL4 in N. benthamiana could induce BAK1- and SOBIR1-dependent cell death, which was dependent on the secretion of SPs. *VdGAL4* is essential for conidial production and morphology, mycelium, microsclerotium, and melanin formation in V. dahliae. In addition, *VdGAL4* regulated the pathogenicity of V. dahliae to cotton by regulating the infection structure of mycelium and the hydrolysis to the cell wall of the host. Collectively, *VdGAL4* played a critical role in fungal development and pathogenicity of V. dahliae as a virulence factor.

## MATERIALS AND METHODS

### Fungal strain, plant material, and culture conditions.

The highly virulent, defoliating V. dahliae strain Vd080 was used as a recipient to generate the different mutant strains in this study. The fungal strains were cultured in potato-dextrose agar (PDA), potato-dextrose broth (PDB), or Czapek-Dox medium unless otherwise specified. The upland cotton (Gossypium hirsutum) cultivar Jimian11, susceptive to V. dahliae, was grown in a greenhouse at 25°C (8 h/16 h dark/light cycle). The culture condition of N. benthamiana is the same as the Jimian11.

### Gene cloning and bioinformatics analysis.

The *VdGAL4* gene was amplified from cDNA or gDNA samples of Vd080 based on the *VdGAL4* sequence in the V. dahliae genome database. The specific primers were designed using Primer5.0 and multiple-sequence alignment using DNAMAN. The signal peptide analysis was implemented using SignalP5.0 (https://services.healthtech.dtu.dk/service.php?SignalP-5.0). The transmembrane domain of VdGAL4 protein was predicted with TMHMM-2.0 (https://services.healthtech.dtu.dk/service.php?TMHMM-2.0). SMART was used to predict the conserved domain of VdGAL4 protein (http://smart.embl.de/smart/set_mode.cgi?NORMAL=1). The primers used are listed in Table S1 in the supplemental material.

### Yeast signal sequence trap system.

Functional verification of predicted signal peptides of VdGAL4 was prepared as described previously (30, 64). The predicted SP sequence of VdGAL4 was amplified with specific primers and fused in-frame to the secretion-defective invertase gene in the pSUC2 vector. The obtained plasmid was transformed into the yeast strain YTK12, and the positive clones were screened on CMD-W medium (deletion of tryptophan). The YTK12 was transformed with pSUC2::Avr1b^SP^ and pSUC2::Mg87^SP^, which were used as positive and negative controls, respectively, and were then incubated in YPRAA medium (2% raffinose) to observe their growth. In addition, the sucrose convertase activity of SP was detected by the reduction of 2,3,5-triphenyltetrazolium chloride (TTC) to an insoluble, red-colored 1,3,5-triphenylformazan (TPF). These transformed yeast strains were cultured in CMD-W medium (30°C, 24 h) and then collected and stained with 2% TTC (30°C, 30 min). Finally, invertase activity was determined by observing and comparing TTC color changes. The primers used are listed in Table S1 in the supplemental material.

### Transient gene expression assays.

The tested genes were amplified from cDNA of Vd080 using the indicated primers, including *VdGAL1*, *VdGAL2*, *VdGAL3*, *VdGAL4*, *VdGAL5*, and the *VdGAL4* without signal peptide (*VdGAL4^ΔSP^*). All of these sequences were cloned severally into the PVX vector pGR107 and transformed into the Agrobacterium tumefaciens strain GV3101. Transient gene expression assays were performed on N. benthamiana plants; Bcl-2-associated X protein (BAX) and green fluorescent protein (GFP) were used as positive and negative controls, respectively (30). All *Agrobacterium* strains were cultured at 28°C for 2 days, resuspended and washed three times, adjusted to an optical density (OD) of 0.8 with infection solution, and then injected into 4-week-old N. benthamiana leaves. Symptom development was monitored from 3 to 7 days postinfiltration. Each assay was performed on 3 leaves from 3 individual plants and repeated 3 times. The primers used are listed in Table S1.

To detect protein expression in N. benthamiana leaves, the total proteins were extracted from the leaves of N. benthamiana plants after *Agrobacterium* infiltration for 48 h. The infiltrated leaves were lysed with radioimmunoprecipitation assay (RIPA) lysis buffer (Beyotime; catalog no. P0013C) at 4°C for 30 min. The lysed samples were centrifuged at 4°C for 10 min, and the supernatants were extracted as the crude protein samples. The protein samples were mixed with protein loading buffer and inactivated at high temperatures for 10 min. The proteins were analyzed by SDS-PAGE and electroporated onto polyvinylidene fluoride (PVDF) membranes (30). Transient protein expression in N. benthamiana was assessed using GFP-tagged polyclonal antibody (Proteintech, China).

### VIGS in N. benthamiana.

For TRV-mediated gene-silencing assays, the plasmid constructs pTRV1, pTRV2-NbBAK1, and pTRV2-NbSOBIR1 were introduced into A. tumefaciens GV3101. The A. tumefaciens strains pTRV1 and pTRV2 were mixed at a ratio of 1: 1, and the final OD_600_ was adjusted to 0.8, which was injected into 3-week-old N. benthamiana leaves (65, 66). pTRV2-PDS was used to evaluate VIGS efficiency, and the pTRV2-GFP was used as control. The RNA extracted from injected leaves was used to validate the efficiency of NbBAK1 or NbSOBIR1 silencing by RT-qPCR. Each assay was performed on 3 leaves from 3 individual plants and repeated 3 times. The primers used are listed in Table S1.

### Subcellular localization assays.

The *VdGAL4* sequence was cloned into the vector pBI121 (pBI121::VdGAL4-RFP) and transformed into the A. tumefaciens strain GV3101. Subsequently, the suspension (OD = 1.0) was injected into N. benthamiana leaves using a previously reported method (67). A. tumefaciens GV3101 carrying pBI121-35S-RFP was used as control. The RFP fluorescence was observed by laser scanning confocal microscope after 36 h. The primers used are listed in Table S1.

### Deletion and complementation of *VdGAL4* in V. dahliae.

Gene deletion and complementation transformants were generated by the A. tumefaciens-mediated transformation (ATMT) method. The *VdGAL4* deletion mutant was constructed using B303 vector. The 1.2-kb upstream fragment and 1.2-kb downstream fragment of *VdGAL4* were separately amplified via PCR with the primer pairs B303-*VdGAL4*-UP-F/R and B303-*VdGAL4*-DOWN-F/R (Table S1). The hygromycin-resistant fragment was amplified from the B303 vector with B303-*VdGAL4*-Hyg-F/R (Table S1). Then, those fragments were connected and introduced into B303 vector by the method of homologous recombination. The positive recombinant vector (B303-*VdGAL4*-UP/Hyg/DOWN) was transferred into A. tumefaciens strain AGL-1 for fungal transformation (68). The positive transformants were screened and isolated in PDA medium containing 50 μg/mL hygromycin for gene deletion and were verified by PCR with the specific primers.

The obtained deletion mutants were further verified by Southern blotting. Southern blot analysis was performed using digoxigenin (DIG) high prime DNA labeling and detection starter kit I (Roche, Germany). Genomic DNA of the WT strain and deletion mutants (*ΔVdGAL4-1* and *ΔVdGAL4-2* strains) was digested by HindIII. A 698-bp *Hyg* fragment (selective marker gene) was used as probe.

The *VdGAL4* complementation mutant was constructed using pSULPH-mut-RG#PB vector. The 1.2-kb upstream fragment and *VdGAL4* genomic DNA were amplified with the primer pairs pSULPH-*VdGAL4*-F/R (Table S1). The positive recombinant vector (pSULPH-*VdGAL4*) was transferred into AGL-1. Subsequently, the obtained deletion mutants were used for fungal transformation of complementation (68). PDA medium containing 100 μg/mL chlorimuron-ethyl was used for complementation mutants screening.

### Enzyme activity and hydrolytic activity of VdGAL4 assays.

For analysis of α-galactosidase activity of VdGAL4, WT, *ΔVdGAL4*, and *C-VdGAL4* strains were incubated in PDB at 25°C for 5 days, and the mycelium was filtered with four layers of gauze and tested with α-galactosidase (α-GAL) activity assay kit (Beijing Boxbio Science & Technology Co., Ltd.). α-Galactosidase can decompose *p*-nitrophenyl-α-d-galactopyranoside into *p*-nitrophenol, so the production of 1 nmol *p*-nitrophenol per gram mycelia per hour is defined as an enzyme activity unit. Experiments were triple replicated.

To analyze the hydrolytic activity of VdGAL4, the conidial suspensions of WT, *ΔVdGAL4*, and *C-VdGAL4* strains were cultured on water agar medium amended with isomaltose or melibiose. After 16 h at 25°C, the dishes were stained with iodine (0.2%)-KI (2%) (volume of 1:1) solution. Experiments were triple replicated.

### Production and phenotype of mycelium and conidia.

For the radial growth rate assay, 5 μL of conidial suspension with a concentration of 5 × 10^6^ conidia/mL of the WT, *ΔVdGAL4*, and *C-VdGAL4* strains was placed in the center of PDA plates and incubated at 25°C for 14 days. Experiments were triple replicated.

To study conidial production of the WT, *ΔVdGAL4*, and *C-VdGAL4* strains, agar plugs were collected using an 8-mm-diameter borer from the edge of mycelium on PDA plates that were cultured for 7 days. Then, the plug was shaken in 1 mL of sterile water, and the number of conidia was quantified under microscope (57). Twenty microliters of the conidial suspension (2 × 10^3^ conidia/mL) was added as a hanging drop to the glass slide and cultured at 25°C for 5 h. The germination rate of conidia was measured with the optical microscope (69). Experiments were triple replicated.

The sterile coverslips were inserted obliquely at the edge of mycelium growth on the PDA plate after 7 days of culture and continued to culture for 2 days. The morphology of mycelium on the coverslips was observed under microscope. The suspensions of high-concentration conidia were collected, and the microscopic morphology of conidia was observed by scanning electron microscope. Experiments were triple replicated.

### Evaluation of microsclerotium formation.

The conidial suspensions (5 × basal modified medium [BMM]; 10^6^ conidia/mL) of the respective strains were evenly spread on a plate of BMM (0.2 g/L NaNO_3_, 0.52 g/L KCl, 0.52 g/L MgSO_4_·7H_2_O, 1.52 g/L KH_2_PO_4_, 3 μmol/L thiamine, 0.1 μmol/L biotin, 5 g/L glucose, and 15 g/L agar, pH 11.5) (70). The plates were incubated at 25°C in the dark for 40 days. Each experiment was triple replicated. The number of microsclerotium was counted by ImageJ.

### Carbon source utilization assays.

For analysis of carbon source utilization, WT, *ΔVdGAL4*, and *C-VdGAL4* strains were cultured in Czapek-Dox medium (without sucrose) amended with different carbon sources (10 g/L pectin, 10 g/L xylan, 10 g/L glucomannan, 10 g/L cellulose, 10 g/L raffinose, 17 g/L starch, and 30 g/L sucrose) (60). Colony morphology was photographed, and diameters were measured after 14 days. Experiments were triple replicated.

### Growth of strains on stress treatments.

The sensitivity of mutants to abiotic stress agents was detected using PDA containing 0.002% SDS, 0.02% Congo red, 1 M sorbitol, 1 M KCl, and 1 M NaCl (49). The relative growth inhibition rate (RGI) was calculated as follows: RGI = (control colony diameter − treatment colony diameter)/control colony diameter × 100% (71). The mycelial growth of WT strains at 25°C without any stress agents was used as the control. In parallel, the sensitivity of mutants to cold, heat, and UV stress was examined. WT, deletion, and complementation strains were cultured with PDA at various temperatures (4°C, 16°C, 25°C, 28°C, 30°C, 33°C) or treated with a 10-s pulse of 302-nm UV light (71). The untreated strains were used as a control. Experiments were triple replicated.

For the thermostability assay, the conidial suspensions (2 × 10^3^ conidia/mL) were heated at 45°C for 1 h and then added as a hanging drop to the glass slide and cultured at 25°C for 5 h (59). The conidial germination efficiency was observed and recorded under microscope. The relative inhibition rate was calculated, and the conidial germination rate of WT strains at 25°C without any stress agents was used as the control. Each experiment was triple replicated.

### Assays for penetration.

The difference in hyphal permeability of WT, *ΔVdGAL4*, and *C-VdGAL4* strains was observed by spreading conidial suspensions (5 × 10^6^ conidia/mL) onto the center of sterilized cellophane membrane covering PDA medium at 25°C for 3 days (57). Then, we removed the cellophane membrane and continue to culture for 3 days. The micromorphology of hyphae on cellophane membrane was observed by scanning electron microscope. Each experiment was triple replicated.

### Assays for pathogenicity and colonization.

Pathogenicity assays were performed on the susceptible cultivar Jimian11 cotton seedlings that were inoculated at the two true leaf stages. The conidial suspensions (5 × 10^6^ conidia/mL) of WT, deletion, and complementation strains were inoculated using a root dip inoculation method (69). Disease severity was recorded at 14, 20, and 26 days postinoculation (dpi) in five categories, and the disease index was calculated as an indicator of the severity of the disease (72). After 26 dpi, the phenotype of cotton plants was recorded, and the vascular discoloration was observed by stereo light microscope. The assay was repeated three times for each strain. At each time for each strain, there were six pots, each with five seedlings.

To qualitatively evaluate the difference in virulence among the WT and mutant strains, the degree of discoloration of vascular tissue was recorded visually on the root and stem that were longitudinally cut. Meanwhile, the stems of the cotton seedlings were cut off, and we placed a stem piece onto PDA plates to isolate any hyphae from vascular bundles (61).

Cotton tissue samples from the stem of inoculated plants were collected at 26 dpi for *in planta* quantification of fungal biomass. The total genomic DNA was extracted with E.Z.N.A. high-performance (HP) fungal DNA kit (Omega Bio-tek) and quantified by NanoDrop 2000, and for each sample, 100 ng of DNA was employed for quantitative real-time PCR (qPCR) reaction. The qPCR was performed using the ChamQ universal SYBR qPCR master mix (Vazyme) with the primers listed in Table S1. V. dahliae β-tubulin (GenBank accession no. DQ266153) was used to quantify fungal colonization. The cotton *UBQ7* gene was used as endogenous plant control. Three independent biological and technical repeats were performed.

### Analysis of gene expression.

Three pathogenic genes related to conidial production, *VdNLP1* (*VDAG_04701.1*), *VdPLP* (*VDAG_00942*), and *Vdpf* (*VDAG_08521.1*), were used for RT-qPCR analysis. For further analyses the effect of *VdGAL4* deficiency on V. dahliae infection structure, such as the infection peg-related genes *VdNoxB* (*VDAG_09930*) and *VdPls1* (*VDAG_01769*); regulating infection peg development of transcription activator *VdCrz1* (*VDAG_03208*); assembling mycelium neck ring-related secretary protein VdSep5 (*VDAG_04382*); regulating appressorium formation-related gene *VdCSIN1* (*VDAG_05652*); and plant root adhesion, penetration, and colonization of V. dahliae-related transcription factors *Som1* (*VDAG_JR2_Chr1g09120a*) and *Vta3* (*VDAG_Chr1g07600a*). To further investigate melanin, microsclerotium formation, and cell wall biogenesis, six genes, including *Vayg1* (*VDAG_04954*), *VT4HR* (*VDAG_03665*), *VaflM* (*VDAG_00183*), *VdSCD* (*VDAG_03393*), *VDH1* (*VDAG_02273*), and *VdLAC* (*VDAG_00189*), were used for RT-qPCR analysis (70).

All of the strains, including WT, deletion, and complementation mutant strains, were cultured in PDA at 25°C for 7 days, and the mycelia were collected after filtering with a 4-layer sponge. Total RNA was extracted with fungal total RNA isolation kit (Sangon Biotech), and cDNA was synthesized with the HiScript II Q RT supermix for qPCR (+genomic DNA [gDNA] wiper) (Vazyme) following the manufacturer’s procedure. The RT-qPCR of the above-described genes (Table S1) was performed with the β-tubulin gene (Table S1) of V. dahliae as an internal reference. The relative expression levels were calculated using the threshold cycle (2^−ΔΔ^*^CT^*) method. The mean and standard error of gene expressions were estimated from three biological replicates.

### Statistical analysis.

Statistical analyses were performed using SPSS statistical software package (v22.0). One-way analysis of variance (ANOVA) was applied and followed by the Student-Newman-Keuls (SNK) test to determine significant differences between treatments at *P* values of 0.05 or 0.01.

## References

[1] Klosterman SJ, Atallah ZK, Vallad GE, Subbarao KV. 2009. Diversity, pathogenicity, and management of *Verticillium* species. Annu Rev Phytopathol 47:39–62. doi:10.1146/annurev-phyto-080508-081748. 19385730

[2] Bhat RG, Subbarao KV. 1999. Host range specificity in *Verticillium dahliae*. Phytopathology 89:1218–1225. doi:10.1094/PHYTO.1999.89.12.1218. 18944648

[3] Tang C, Xiong D, Fang Y, Tian C, Wang Y. 2017. The two-component response regulator *VdSkn7* plays key roles in microsclerotial development, stress resistance and virulence of *Verticillium dahliae*. Fungal Genet Biol 108:26–35. doi:10.1016/j.fgb.2017.09.002. 28917999

[4] Fan R, Klosterman SJ, Wang C, Subbarao KV, Xu X, Shang W, Hu X. 2017. *Vayg1* is required for microsclerotium formation and melanin production in *Verticillium dahliae*. Fungal Genet Biol 98:1–11. doi:10.1016/j.fgb.2016.11.003. 27866941

[5] Fradin EF, Thomma BP. 2006. Physiology and molecular aspects of Verticillium wilt diseases caused by *V. dahliae* and *V. albo-atrum*. Mol Plant Pathol 7:71–86. doi:10.1111/j.1364-3703.2006.00323.x. 20507429

[6] Lo Presti L, Lanver D, Schweizer G, Tanaka S, Liang L, Tollot M, Zuccaro A, Reissmann S, Kahmann R. 2015. Fungal effectors and plant susceptibility. Annu Rev Plant Biol 66:513–545. doi:10.1146/annurev-arplant-043014-114623. 25923844

[7] Zhao P, Zhao YL, Jin Y, Zhang T, Guo HS. 2014. Colonization process of *Arabidopsis thaliana* roots by a green fluorescent protein-tagged isolate of *Verticillium dahliae*. Protein Cell 5:94–98. doi:10.1007/s13238-013-0009-9. 24481631 PMC3956967

[8] Boller T, Felix G. 2009. A renaissance of elicitors: perception of microbe-associated molecular patterns and danger signals by pattern-recognition receptors. Annu Rev Plant Biol 60:379–406. doi:10.1146/annurev.arplant.57.032905.105346. 19400727

[9] Roux M, Schwessinger B, Albrecht C, Chinchilla D, Jones A, Holton N, Malinovsky FG, Tor M, de Vries S, Zipfel C. 2011. The *Arabidopsis* leucine-rich repeat receptor–like kinases BAK1/SERK3 and BKK1/SERK4 are required for innate immunity to hemibiotrophic and biotrophic pathogens. Plant Cell 23:2440–2455. doi:10.1105/tpc.111.084301. 21693696 PMC3160018

[10] Liebrand TWH, van den Berg GCM, Zhang Z, Smit P, Cordewener JHG, America AHP, America AHP, Sklenar J, Jones AME, Tameling WIL, Robatzek S, Thomma BPHJ, Joosten MHAJ. 2013. Receptor-like kinase SOBIR1/EVR interacts with receptor-like proteins in plant immunity against fungal infection. Proc Natl Acad Sci USA 110:10010–10015. doi:10.1073/pnas.1220015110. 23716655 PMC3683720

[11] Bui TT, Harting R, Braus-Stromeyer SA, Tran VT, Leonard M, Hofer A, Abelmann A, Bakti F, Valerius O, Schluter R, Stanley CE, Ambrosio A, Braus GH. 2019. *Verticillium dahliae* transcription factors Som1 and Vta3 control microsclerotia formation and sequential steps of plant root penetration and colonisation to induce disease. New Phytol 221:2138–2159. doi:10.1111/nph.15514. 30290010

[12] Ma L, Cornelissen BJ, Takken FL. 2013. A nuclear localization for Avr2 from *Fusarium oxysporum* is required to activate the tomato resistance protein I-2. Front Plant Sci 4:94. doi:10.3389/fpls.2013.00094. 23596453 PMC3622885

[13] Gui YJ, Chen JY, Zhang DD, Li NY, Li TG, Zhang WQ, Wang XY, Short DPG, Li L, Guo W, Kong ZQ, Bao YM, Subbarao KV, Dai XF. 2017. *Verticillium dahliae* manipulates plant immunity by glycoside hydrolase 12 proteins in conjunction with carbohydrate-binding module 1. Environ Microbiol 19:1914–1932. doi:10.1111/1462-2920.13695. 28205292

[14] Zheng J, Tang C, Deng C, Wang Y. 2019. Involvement of a response regulator *VdSsk1* in stress response, melanin biosynthesis and full virulence in *Verticillium dahliae*. Front Microbiol 10:606. doi:10.3389/fmicb.2019.00606. 30967857 PMC6439524

[15] Wang H, Chen B, Tian J, Kong Z. 2021. *Verticillium dahliae* VdBre1 is required for cotton infection by modulating lipid metabolism and secondary metabolites. Environ Microbiol 23:1991–2003. doi:10.1111/1462-2920.15319. 33185953

[16] Zhang YL, Li ZF, Feng ZL, Feng HJ, Shi YQ, Zhao LH, Zhang XL, Zhu HQ. 2016. Functional analysis of the pathogenicity-related gene *VdPR1* in the vascular wilt fungus *Verticillium dahliae*. PLoS One 11:e0166000. doi:10.1371/journal.pone.0166000. 27846253 PMC5112940

[17] Zhang YL, Li ZF, Feng ZL, Feng HJ, Zhao LH, Shi YQ, Hu XP, Zhu HQ. 2015. Isolation and functional analysis of the pathogenicity-related gene *VdPR3* from *Verticillium dahliae* on cotton. Curr Genet 61:555–566. doi:10.1007/s00294-015-0476-z. 25652159

[18] Zhang J, Cui W, Abdul Haseeb H, Guo W. 2020. *VdNop12*, containing two tandem RNA recognition motif domains, is a crucial factor for pathogenicity and cold adaption in *Verticillium dahliae*. Environ Microbiol 22:5387–5401. doi:10.1111/1462-2920.15268. 33000558

[19] Qin T, Hao W, Sun R, Li Y, Wang Y, Wei C, Dong T, Wu B, Dong N, Wang W, Sun J, Yang Q, Zhang Y, Yang S, Wang Q. 2020. *Verticillium dahliae* VdTHI20, involved in pyrimidine biosynthesis, is required for DNA repair functions and pathogenicity. Int J Mol Sci 21:1378. doi:10.3390/ijms21041378. 32085660 PMC7073022

[20] Xiong D, Wang Y, Tang C, Fang Y, Zou J, Tian C. 2015. *VdCrz1* is involved in microsclerotia formation and required for full virulence in *Verticillium dahliae*. Fungal Genet Biol 82:201–212. doi:10.1016/j.fgb.2015.07.011. 26235044

[21] Luo X, Mao H, Wei Y, Cai J, Xie C, Sui A, Yang X, Dong J. 2016. The fungal-specific transcription factor *Vdpf* influences conidia production, melanized microsclerotia formation and pathogenicity in *Verticillium dahliae*. Mol Plant Pathol 17:1364–1381. doi:10.1111/mpp.12367. 26857810 PMC6638448

[22] Ma Z, Song T, Zhu L, Ye W, Wang Y, Shao Y, Dong S, Zhang Z, Dou D, Zheng X, Tyler BM, Wang Y. 2015. A *Phytophthora sojae* glycoside hydrolase 12 protein is a major virulence factor during soybean infection and is recognized as a PAMP. Plant Cell 27:2057–2072. doi:10.1105/tpc.15.00390. 26163574 PMC4531360

[23] Ma Z, Zhu L, Song T, Wang Y, Zhang Q, Xia Y, Qiu M, Lin Y, Li H, Kong L, Fang Y, Ye W, Wang Y, Dong S, Zheng X, Tyler BM, Wang Y. 2017. A paralogous decoy protects *Phytophthora sojae* apoplastic effector PsXEG1 from a host inhibitor. Science 355:710–714. doi:10.1126/science.aai7919. 28082413

[24] Zhang L, Yan J, Fu Z, Shi W, Ninkuu V, Li G, Yang X, Zeng H. 2021. FoEG1, a secreted glycoside hydrolase family 12 protein from *Fusarium oxysporum*, triggers cell death and modulates plant immunity. Mol Plant Pathol 22:522–538. doi:10.1111/mpp.13041. 33675158 PMC8035634

[25] Liu L, Wang Z, Li J, Wang Y, Yuan J, Zhan J, Wang P, Lin Y, Li F, Ge X. 2021. *Verticillium dahliae* secreted protein Vd424Y is required for full virulence, targets the nucleus of plant cells, and induces cell death. Mol Plant Pathol 22:1109–1120. doi:10.1111/mpp.13100. 34233072 PMC8358993

[26] Butler MJ, Day AW. 1998. Fungal melanins: a review. Can J Microbiol 44:1115–1136. doi:10.1139/w98-119.

[27] Weignerová L, Simerská P, Křen V. 2009. α-Galactosidases and their applications in biotransformations. Biocatal Biotransformation 27:79–89. doi:10.1080/10242420802583416.

[28] Wang D, Chen JY, Song J, Li JJ, Klosterman SJ, Li R, Kong ZQ, Subbarao KV, Dai XF, Zhang DD. 2021. Cytotoxic function of xylanase VdXyn4 in the plant vascular wilt pathogen *Verticillium dahliae*. Plant Physiol 187:409–429. doi:10.1093/plphys/kiab274. 34618145 PMC8418393

[29] Zhang X, Zhao L, Liu S, Zhou J, Wu Y, Feng Z, Zhang Y, Zhu H, Wei F, Feng H. 2022. Identification and functional analysis of a novel hydrophobic protein VdHP1 from *Verticillium dahliae*. Microbiol Spectr 10:e0247821. doi:10.1128/spectrum.02478-21. 35377232 PMC9045179

[30] Wang D, Zhang DD, Song J, Li JJ, Wang J, Li R, Klosterman SJ, Kong ZQ, Lin FZ, Dai XF, Subbarao KV, Chen JY. 2022. *Verticillium dahliae* CFEM proteins manipulate host immunity and differentially contribute to virulence. BMC Biol 20:55. doi:10.1186/s12915-022-01254-x. 35197059 PMC8867779

[31] Xia Y, Ma Z, Qiu M, Guo B, Zhang Q, Jiang H, Zhang B, Lin Y, Xuan M, Sun L, Shu H, Xiao J, Ye W, Wang Y, Wang Y, Dong S, Tyler BM, Wang Y. 2020. N-glycosylation shields *Phytophthora sojae* apoplastic effector PsXEG1 from a specific host aspartic protease. Proc Natl Acad Sci USA 117:27685–27693. doi:10.1073/pnas.2012149117. 33082226 PMC7959567

[32] Fujimoto Z, Kaneko S, Momma M, Kobayashi H, Mizuno H. 2003. Crystal structure of rice alpha-galactosidase complexed with D-galactose. J Biol Chem 278:20313–20318. doi:10.1074/jbc.M302292200. 12657636

[33] Bhatia S, Singh A, Batra N, Singh J. 2020. Microbial production and biotechnological applications of alpha-galactosidase. Int J Biol Macromol 150:1294–1313. doi:10.1016/j.ijbiomac.2019.10.140. 31747573

[34] Alvarez-Cao ME, Cerdan ME, Gonzalez-Siso MI, Becerra M. 2019. Optimization of *Saccharomyces cerevisiae* alpha-galactosidase production and application in the degradation of raffinose family oligosaccharides. Microb Cell Fact 18:172. doi:10.1186/s12934-019-1222-x. 31601209 PMC6786279

[35] Wang H, Luo H, Li J, Bai Y, Huang H, Shi P, Fan Y, Yao B. 2010. An alpha-galactosidase from an acidophilic *Bispora* sp. MEY-1 strain acts synergistically with beta-mannanase. Bioresour Technol 101:8376–8382. doi:10.1016/j.biortech.2010.06.045. 20591661

[36] Chroumpi T, Martinez-Reyes N, Kun RS, Peng M, Lipzen A, Ng V, Tejomurthula S, Zhang Y, Grigoriev IV, Makela MR, de Vries RP, Garrigues S. 2022. Detailed analysis of the D-galactose catabolic pathways in *Aspergillus niger* reveals complexity at both metabolic and regulatory level. Fungal Genet Biol 159:103670. doi:10.1016/j.fgb.2022.103670. 35121171

[37] Arunraj R, Skori L, Kumar A, Hickerson NMN, Shoma N, Vairamani M, Samuel MA. 2020. Spatial regulation of alpha-galactosidase activity and its influence on raffinose family oligosaccharides during seed maturation and germination in Cicer arietinum. Plant Signal Behav 15:1709707. doi:10.1080/15592324.2019.1709707. 31906799 PMC8570745

[38] Xiong D, Wang Y, Tian L, Tian C. 2016. MADS-box transcription factor VdMcm1 regulates conidiation, microsclerotia formation, pathogenicity, and secondary metabolism of *Verticillium dahliae*. Front Microbiol 7:1192. doi:10.3389/fmicb.2016.01192. 27536281 PMC4971026

[39] Luo XM, Xie CJ, Dong JY, Yang XY, Sui AP. 2014. Interactions between *Verticillium dahliae* and its host: vegetative growth, pathogenicity, plant immunity. Appl Microbiol Biotechnol 98:6921–6932. doi:10.1007/s00253-014-5863-8. 24928658

[40] Klimes A, Amyotte SG, Grant S, Kang S, Dobinson KF. 2008. Microsclerotia development in *Verticillium dahliae*: regulation and differential expression of the hydrophobin gene *VDH1*. Fungal Genet Biol 45:1525–1532. doi:10.1016/j.fgb.2008.09.014. 18951989

[41] Klimes A, Dobinson KF. 2006. A hydrophobin gene, *VDH1*, is involved in microsclerotial development and spore viability in the plant pathogen *Verticillium dahliae*. Fungal Genet Biol 43:283–294. doi:10.1016/j.fgb.2005.12.006. 16488633

[42] Zhou BJ, Jia PS, Gao F, Guo HS. 2012. Molecular characterization and functional analysis of a necrosis- and ethylene-inducing, protein-encoding gene family from *Verticillium dahliae*. Mol Plant Microbe Interact 25:964–975. doi:10.1094/MPMI-12-11-0319. 22414440

[43] Gao F, Zhou BJ, Li GY, Jia PS, Li H, Zhao YL, Zhao P, Xia GX, Guo HS. 2010. A glutamic acid-rich protein identified in *Verticillium dahliae* from an insertional mutagenesis affects microsclerotial formation and pathogenicity. PLoS One 5:e15319. doi:10.1371/journal.pone.0015319. 21151869 PMC2998422

[44] Eisenman HC, Greer EM, McGrail CW. 2020. The role of melanins in melanotic fungi for pathogenesis and environmental survival. Appl Microbiol Biotechnol 104:4247–4257. doi:10.1007/s00253-020-10532-z. 32206837

[45] Luo X, Xie C, Dong J, Yang X. 2019. Comparative transcriptome analysis reveals regulatory networks and key genes of microsclerotia formation in the cotton vascular wilt pathogen. Fungal Genet Biol 126:25–36. doi:10.1016/j.fgb.2019.01.009. 30710746

[46] Li H, Wang D, Zhang DD, Geng Q, Li JJ, Sheng RC, Xue HS, Zhu H, Kong ZQ, Dai XF, Klosterman SJ, Subbarao KV, Chen FM, Chen JY. 2022. A polyketide synthase from *Verticillium dahliae* modulates melanin biosynthesis and hyphal growth to promote virulence. BMC Biol 20:125. doi:10.1186/s12915-022-01330-2. 35637443 PMC9153097

[47] Lai M, Cheng Z, Xiao L, Klosterman SJ, Wang Y. 2022. The bZip transcription factor *VdMRTF1* is a negative regulator of melanin biosynthesis and virulence in *Verticillium dahliae*. Microbiol Spectr 10:e0258121. doi:10.1128/spectrum.02581-21. 35404080 PMC9045294

[48] Tian L, Wang Y, Yu J, Xiong D, Zhao H, Tian C. 2016. The mitogen-activated protein kinase VdPbs2 of *Verticillium dahliae* regulates microsclerotia formation, stress response, and plant infection. Front Microbiol 7:1532. doi:10.3389/fmicb.2016.01532. 27729908 PMC5037172

[49] Yu J, Li T, Tian L, Tang C, Klosterman SJ, Tian C, Wang Y. 2019. Two *Verticillium dahliae* MAPKKKs, *VdSsk2* and *VdSte11*, have distinct roles in pathogenicity, microsclerotial formation, and stress adaptation. mSphere 4:e00426-19. doi:10.1128/mSphere.00426-19. 31292234 PMC6620378

[50] Lorrai R, Ferrari S. 2021. Host cell wall damage during pathogen infection: mechanisms of perception and role in plant-pathogen interactions. Plants (Basel) 10:399. doi:10.3390/plants10020399. 33669710 PMC7921929

[51] Wang H, Wang J, Mujumdar AS, Jin XW, Liu ZL, Zhang Y, Xiao HW. 2021. Effects of postharvest ripening on physicochemical properties, microstructure, cell wall polysaccharides contents (pectin, hemicellulose, cellulose) and nanostructure of kiwifruit (*Actinidia deliciosa*). Food Hydrocoll 118:106808. doi:10.1016/j.foodhyd.2021.106808.

[52] Xiao R, Zhang C, Guo X, Li H, Lu H. 2021. MYB transcription factors and its regulation in secondary cell wall formation and lignin biosynthesis during xylem development. Int J Mol Sci 22:3560. doi:10.3390/ijms22073560. 33808132 PMC8037110

[53] Vorwerk S, Somerville S, Somerville C. 2004. The role of plant cell wall polysaccharide composition in disease resistance. Trends Plant Sci 9:203–209. doi:10.1016/j.tplants.2004.02.005. 15063871

[54] Białas A, Zess EK, De la Concepcion JC, Franceschetti M, Pennington HG, Yoshida K, Upson JL, Chanclud E, Wu C-H, Langner T, Maqbool A, Varden FA, Derevnina L, Belhaj K, Fujisaki K, Saitoh H, Terauchi R, Banfield MJ, Kamoun S. 2018. Lessons in effector and NLR biology of plant-microbe systems. Mol Plant Microbe Interact 31:34–45. doi:10.1094/MPMI-08-17-0196-FI. 29144205

[55] Shi Y, Wang H, Yan Y, Cao H, Liu X, Lin F, Lu J. 2018. Glycerol-3-phosphate shuttle is involved in development and virulence in the rice blast fungus *Pyricularia oryzae*. Front Plant Sci 9:687. doi:10.3389/fpls.2018.00687. 29875789 PMC5974175

[56] Li X, Su X, Lu G, Sun G, Zhang Z, Guo H, Guo N, Cheng H. 2020. *VdOGDH* is involved in energy metabolism and required for virulence of *Verticillium dahliae*. Curr Genet 66:345–359. doi:10.1007/s00294-019-01025-2. 31422448

[57] Tian L, Li J, Huang C, Zhang D, Xu Y, Yang X, Song J, Wang D, Qiu N, Short DPG, Inderbitzin P, Subbarao KV, Chen J, Dai X. 2021. Cu/Zn superoxide dismutase (VdSOD1) mediates reactive oxygen species detoxification and modulates virulence in *Verticillium dahliae*. Mol Plant Pathol 22:1092–1108. doi:10.1111/mpp.13099. 34245085 PMC8359004

[58] Singh LP, Gill SS, Tuteja N. 2011. Unraveling the role of fungal symbionts in plant abiotic stress tolerance. Plant Signal Behav 6:175–191. doi:10.4161/psb.6.2.14146. 21512319 PMC3121976

[59] Zhang S, Xia YX, Kim B, Keyhani NO. 2011. Two hydrophobins are involved in fungal spore coat rodlet layer assembly and each play distinct roles in surface interactions, development and pathogenesis in the entomopathogenic fungus, *Beauveria bassiana*. Mol Microbiol 80:811–826. doi:10.1111/j.1365-2958.2011.07613.x. 21375591

[60] Qi X, Su X, Guo H, Qi J, Cheng H. 2016. VdThit, a thiamine transport protein, is required for pathogenicity of the vascular pathogen *Verticillium dahliae*. Mol Plant Microbe Interact 29:545–559. doi:10.1094/MPMI-03-16-0057-R. 27089469

[61] Zhao YL, Zhou TT, Guo HS. 2016. Hyphopodium-specific *VdNoxB*/*VdPls1*-dependent ROS-Ca^2+^ signaling is required for plant infection by *Verticillium dahliae*. PLoS Pathog 12:e1005793. doi:10.1371/journal.ppat.1005793. 27463643 PMC4962994

[62] Kawamura C, Moriwaki J, Kimura N, Fujita Y, Fuji S, Hirano T, Koizumi S, Tsuge T. 1997. The melanin biosynthesis genes of *Alternaria alternata* can restore pathogenicity of the melanin-deficient mutants of *Magnaporthe grisea*. Mol Plant Microbe Interact 10:446–453. doi:10.1094/MPMI.1997.10.4.446. 9150594

[63] Zhang J, Zhang Y, Yang J, Kang L, Elo RA, Zhou H, Zhao J. 2019. The alpha-1,6-mannosyltransferase VdOCH1 plays a major role in microsclerotium formation and virulence in the soil-borne pathogen *Verticillium dahliae*. Fungal Biol 123:539–546. doi:10.1016/j.funbio.2019.05.007. 31196523

[64] Yin WX, Wang YF, Chen T, Lin Y, Luo CX. 2018. Functional evaluation of the signal peptides of secreted proteins. Bio-Protocol 8:e2839. doi:10.21769/BioProtoc.2839. 34286044 PMC8275294

[65] Nie JJ, Yin ZY, Li ZP, Wu YX, Huang LL. 2019. A small cysteine-rich protein from two kingdoms of microbes is recognized as a novel pathogen-associated molecular pattern. New Phytol 222:995–1011. doi:10.1111/nph.15631. 30537041

[66] Yin CM, Li JJ, Wang D, Zhang DD, Song J, Kong ZQ, Wang BL, Hu XP, Klosterman SJ, Subbarao KV, Chen JY, Dai XF. 2022. A secreted ribonuclease effector from *Verticillium dahliae* localizes in the plant nucleus to modulate host immunity. Mol Plant Pathol 23:1122–1140. doi:10.1111/mpp.13213. 35363930 PMC9276946

[67] Feng H, Li C, Zhou J, Yuan Y, Feng Z, Shi Y, Zhao L, Zhang Y, Wei F, Zhu H. 2021. A cotton WAKL protein interacted with a DnaJ protein and was involved in defense against *Verticillium dahliae*. Int J Biol Macromol 167:633–643. doi:10.1016/j.ijbiomac.2020.11.191. 33275973

[68] Liu SY, Chen JY, Wang JL, Li L, Xiao HL, Adam SM, Dai XF. 2013. Molecular characterization and functional analysis of a specific secreted protein from highly virulent defoliating *Verticillium dahliae*. Gene 529:307–316. doi:10.1016/j.gene.2013.06.089. 23891822

[69] Zhou J, Feng Z, Liu S, Wei F, Shi Y, Zhao L, Huang W, Zhou Y, Feng H, Zhu H. 2021. CGTase, a novel antimicrobial protein from *Bacillus cereus* YUPP-10, suppresses *Verticillium dahliae* and mediates plant defence responses. Mol Plant Pathol 22:130–144. doi:10.1111/mpp.13014. 33230892 PMC7749748

[70] Hu D, Wang C, Tao F, Cui Q, Xu X, Shang W, Hu X. 2014. Whole genome wide expression profiles on germination of *Verticillium dahliae* microsclerotia. PLoS One 9:e100046. doi:10.1371/journal.pone.0100046. 24927478 PMC4057337

[71] Liu J, Wang ZK, Sun HH, Ying SH, Feng MG. 2017. Characterization of the Hog1 MAPK pathway in the entomopathogenic fungus *Beauveria bassiana*. Environ Microbiol 19:1808–1821. doi:10.1111/1462-2920.13671. 28076898

[72] Zhu D, Zhang X, Zhou J, Wu Y, Zhang X, Feng Z, Wei F, Zhao L, Zhang Y, Shi Y, Feng H, Zhu H. 2021. Genome-wide analysis of ribosomal protein GhRPS6 and its role in cotton Verticillium wilt resistance. Int J Mol Sci 22:1795. doi:10.3390/ijms22041795. 33670294 PMC7918698

