# Supplementary material for: VdGAL4 Modulates Microsclerotium Formation, Conidial Morphology, and Germination To Promote Virulence in Verticillium dahliae
Source: Microbiol Spectr. 2022 Dec 7;11(1):e03515-22. doi: 10.1128/spectrum.03515-22 (PMC9927093; doi:10.1128/spectrum.03515-22)
Supplement: Supplemental file 1 — Fig. S1 to S4 and Table S1. Download spectrum.03515-22-s0001.pdf, PDF file, 0.5 MB [file spectrum.03515-22-s0001.pdf]

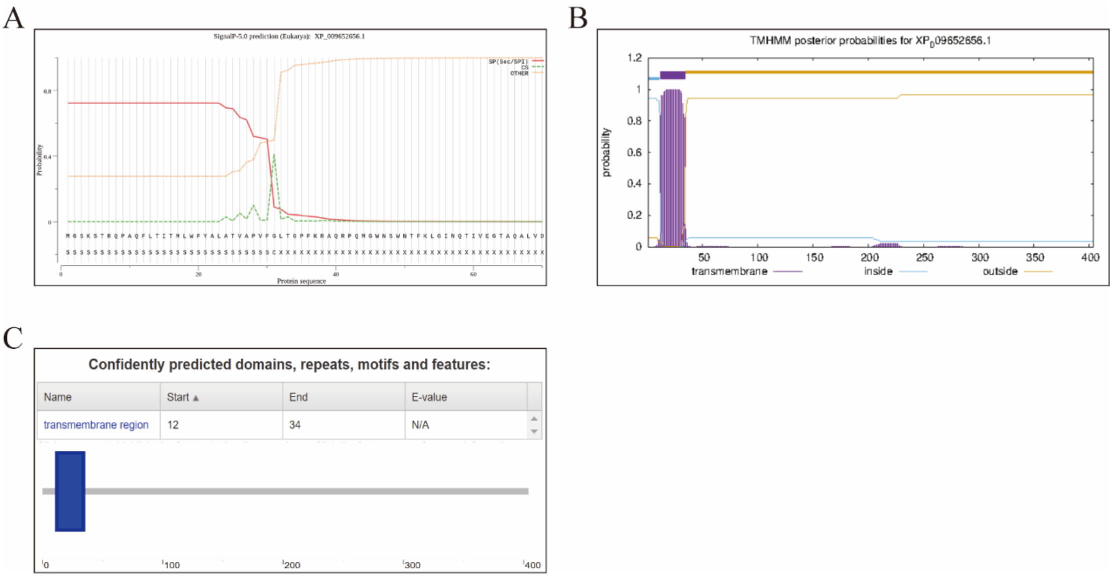

FIG S1 Bioinformatics analysis of VdGAL4. (A) The signal peptide sites of VdGAL4 protein. (B) Transmembrane domain analysis of VdGAL4. (C) The conserved domain of VdGAL4.

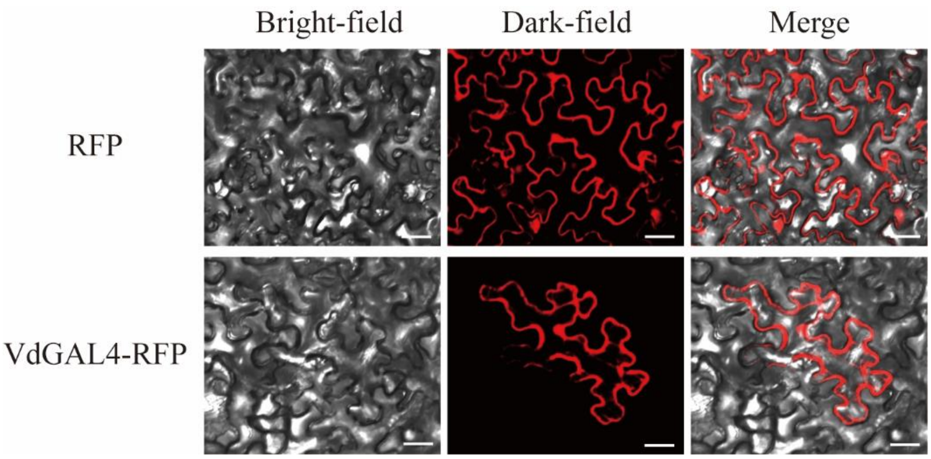

FIG S2 Subcellular localizations of VdGAL4 in *N. benthamiana* on *A. tumefaciens*-mediated transient expression. Fluorescence was detected by confocal microscopy after 48 h of injection. Scale bar = 100 µm.

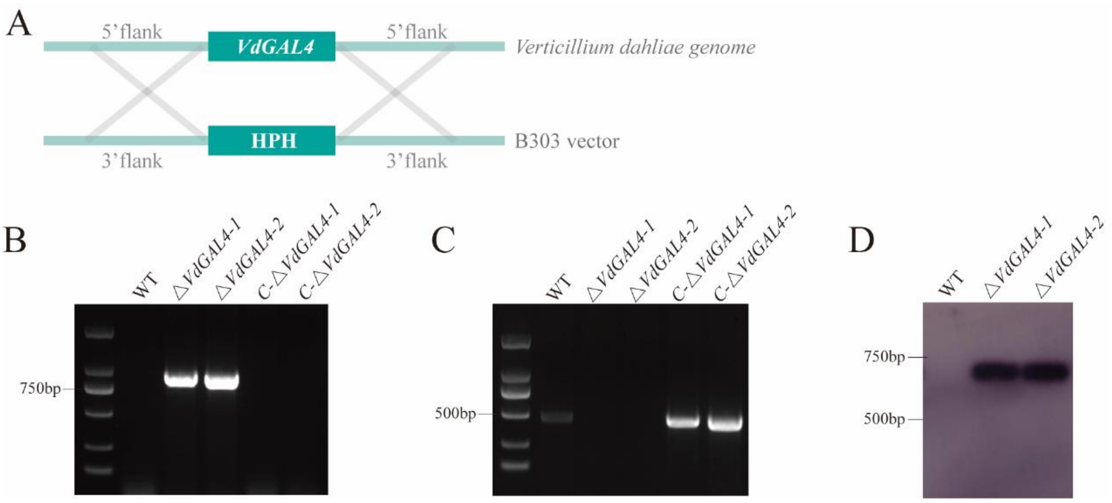

FIG S3 Identification of deletion and complementary mutants. (A) The principle of obtaining the mutant of *VdGAL4* by homologous recombination method. (B) The deletion mutant strains of *VdGAL4* were determined by PCR. (C) The complementary mutant strains of *VdGAL4* were determined by PCR. (D) Southern blotting was used to identify the deletion mutant strains.

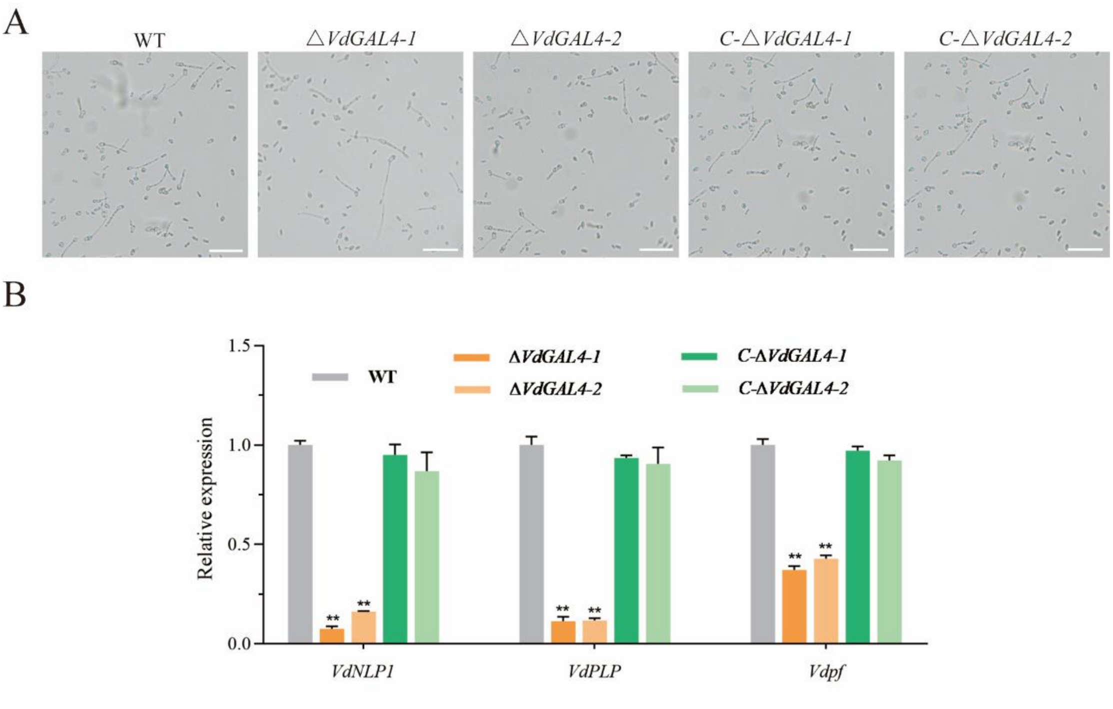

FIG S4 (A) The conidia of WT, *ΔVdGAL4* and *C-ΔVdGAL4* strains germinated at 25°C. Bar = 200 μm. (B) Assays of the relative expression of conidia-related genes in all tested strains by RT-qPCR. The error bar represents standard error of means. $*p < 0.05$, $**p < 0.01$.

Table S1： Primers used in this study.

Primer name	Sequence (5’-3’)	Enzyme loci	Purpose
pGR107-VdGAL1-F	GGTCAGCACCAGCTAGCATCGATATGGGTGTCATTCGCAAC	Cla I	Transient gene expression
pGR107-VdGAL1-R	TCGCCCTTGCTCACCATCCCGGGCTCGGTCTCCTGATCAGCCAG	Smal I	
pGR107-VdGAL2-F	GGTCAGCACCAGCTAGCATCGATATGCCTTCCAACACAGCG	Cla I	
pGR107-VdGAL2-R	TCGCCCTTGCTCACCATCCCGGGCGCATAAGTCGCGAAGCCTAC	Smal I	
pGR107-VdGAL3-F	GGTCAGCACCAGCTAGCATCGATATGGCACCACTTGCCACA	Cla I	
pGR107-VdGAL3-R	TCGCCCTTGCTCACCATCCCGGGCAGCTCCTCCTTGGTCATGGA	Smal I	
pGR107-VdGAL4-F	GGTCAGCACCAGCTAGCATCGATATGGGTTCAAAGTCCACT	Cla I	
pGR107-VdGAL4-R	TCGCCCTTGCTCACCATCCCGGGGCAAGAAAGCAGCAACACCTT	Smal I	
pGR107-VdGAL5-F	GGTCAGCACCAGCTAGCATCGATATGGCCCGACACTCCACC	Cla I	
pGR107-VdGAL5-R	TCGCCCTTGCTCACCATCCCGGGCAGCTCATCCTTTTCCTCGCG	Smal I	
pGR107-VdGAL4(Δ SP)-F	GGTCAGCACCAGCTAGCATCGATATGACTGACCGGTCCTTTC	Cla I	
pGR107-VdGAL4(Δ SP)-R	TCGCCCTTGCTCACCATCCCGGGGCAAGAAAGCAGCAACACCTT	Smal I	
pSUC2-VdGAL4(SP)-F	CCGGAATTCATGGGTTCAAAGTCC	EcoR I	Yeast signal sequence trap system
pSUC2-VdGAL4(SP)-R	CCGCTCGAGCCCGAAAACGGGAGC	Xho I	
PBI121(RFP)-VdGAL4-F	AGAACACGGGGGACTCTAGAATGAAATTTACCGGCCTTGCTTCG	Xba I	Subcellular localization
PBI121(RFP)-VdGAL4-R	GTTTTGTTAATTAAGGATCCGAAGGTCAGCGTCTGGCC	BamH I	
TRV-BAK1-F	CGGGATCCGTGAGGGTGGTGAGCGGGATAAT		VIGS in *N. benthamiana*
TRV-BAK1-R	CGGAATTCGCTCATAACTGGGCAAAGGGCTT		
TRV-SOBIR1-F	CGGGATCCAATCTTTATCCACCAGATCATGC		
TRV-SOBIR1-R	CGGAATTCCAGAAAGTTTTCCAATGGCAG		

qPCR-*NbActin*-F	TGGTCGTACCACCGGTATTGTGTT		
qPCR-*NbActin*-R	TCACTTGCCCATCAGGAAGCTCAT		
qPCR-*NbBAK1*-F	GAGGTGGGAGGAATGGCAAA		
qPCR-*NbBAK1*-R	TTGGCCCCGACAATTCATCT		
qPCR-*NbSOBIR1*-F	CCAGCAAGTCACAGAAGGGA		
qPCR-*NbSOBIR1*-R	CCAACACCACACCAAAGCTG		
B303-*VdGAL4*-UP-F	GGACCGGACGGGGCGGTACCCCAGGATTC GATCAATAATAT	Kpn I	Generation of *VdGAL4* deletion mutant in *V. dahliae*
B303-*VdGAL4*-UP-R	CTTCAATATCAGTTAACGTCTTTCTCCTTGTT CAAAGTCCA		
B303-*VdGAL4*-Hyg-F	TGGACTTTGAACAAGGAGAAAGACGTTAAC TGATATTGAAG		
B303-*VdGAL4*-Hyg-R	CTGACTGAATTATTTCCTTTACTATTCCTTTG CCCTCGGACG		
B303-*VdGAL4*-Down-F	CGTCCGAGGGCAAAGGAATAGTAAAGGAA ATAATTCAGTCAG		
B303-*VdGAL4*-Down-R	TAGTCCCGGGTCTTAATTAACGTGAAAGACC AGCCAATGGC	Pac I	
pSULPH- *VdGAL4*(+UP)-F	ACGGCCAGTGCCAAGCTTCCAGGATTCGAT CAATAA	BamH I	Generation of *VdGAL4* complementation mutant in *V. dahliae*
pSULPH-*VdGAL4*(+UP)-R	ATTCACTAGTCAGGATCCCTGCAAGAAAGC AGCAACACCTTG	Hind III	
Test-Hyg-F	TCGTTATGTTTATCGGCACT		Validation of *VdGAL4* deletion and complementation mutant in *V. dahlia*
Test-Hyg-F	TCGGTCGGCATCTACTCT		
Test-*VdGAL4*-F	GGACATCCACGCCAAGTT		
Test-*VdGAL4*-R	TCAGAGCAAGCGCAACAT		
Test-Hyg- southern -F	TCGCCCTTCCTCCCTTTA		
Test-Hyg- southern -R	CGCTGTTATGCGGCCATT		
β-tubulin-F	TCACCAGCCGTGGCAAGGTTG		Quantification of fungal biomass
β-tubulin-R	AGCAAAGGGCGGTCTGGACGTTG		
GhUBQ7-F	GAAGGCATTCCACCTGACCAAC		
GhUBQ7-R	CTTGACCTTCTTCTTCTTGTGCTTG		
VdNLP1-F	TCGGTCTTTGCCCTCGTC		qPCR of genes

VdNLP1-R	GCCTGGTTTGCGTTGTTC	related conidial production
VdPLP-F	GCTGACCAGTATCTGTCGGAGG	
VdPLP-R	ATGACGACTGGCTTCTCGGCCT	
Vdpf-F	ACCATTTTCAACAGTCGGGTACGCG	
Vdpf-R	ACCATTTTCAACAGTCGGGTACGCG	
VdNoxB-F	TGCGTGGCAAGCATAAGACATAC	qPCR of genes related mycelial penetration
VdNoxB-R	GACAGCACGAGTGAAATCACCAAC	
VdPls1-F	ATGGTCAACAAGATCCTCGCGA	
VdPls1-R	TCCGGCTGCTCAAACATGTTGT	
VdSep5-F	AGCTCGACCTGGACGAGGA	
VdSep5-R	GAGGCTTCGTTATCAATCTCGTCTC	
VdCrz1-F	ATGGATCAGCAAGCTCAACATCG	
VdCrz1-R	GATCCAGACCGAGACCGAGAC	
VdCSIN1-F	CTTTGATTGTGGTATGGGTTCT	
VdCSIN1-R	GTGGTGGGTTTGCCTTGT	
Som1-F	GTCGTGACAACCGAAAGCAG	
Som1-R	TCCCTCGTGGAGCGCAAA	
Vta3-F	GATGTCTGCCCTGCGTAA	
Vta3-R	GATCTGAGCCTGGTCAAAGT	
Vayg1-F	GTTGCGACGAGTTCTTGT	qPCR of genes related melanin formation
Vayg1-R	ACCATCACCTTGCCCATA	
VT4HR-F	TGGTGGCATCAAGACAGACA	
VT4HR-R	CGAAGCGAGGAAGCAAACAA	
VaflM-F	GACTGTCAATGCCATCGCC	
VaflM-R	CGGTGACCTTGATAACTT	
VdSCD-F	ATGCCCGCTTCCGAGTTC	

VdSCD-R	TTCCACACGCCGTCAATCTT
VDH1-F	GTCTATTCATCTGGTTCCTCCCTA
VDH1-R	CAAACCTCTTACAATGTTGACGC
VdLAC-F	CGTTTCCTCACTTTAGCCACAGC
VdLAC-R	CACCCAGTCCACCGTCCATTTGT
